# Ideologies toward CSL: SL perceptions in the 2025 Chinese Spring Festival Gala

**DOI:** 10.1093/jdsade/enag016

**Published:** 2026-05-20

**Authors:** Li Chuqiao

**Affiliations:** University of Edinburgh, United Kingdom

## Abstract

China has been working on the Sign Language (SL) Translation of TV programs. Based on Loh's (2025a) theory of language ideology, this paper uses the accessible retransmission of the 2025 China Media Group Spring Festival Gala as a case study to explore online perceptions of Chinese Sign Language (CSL) among deaf and hearing Chinese. It combines quantitative analysis of public online comments about the CSL interpretation during the Gala with qualitative analysis of online feedback from deaf Chinese. This study finds that although the novel SL Translation improved the public’s impression of CSL and consideration of deaf people, issues of Chinese barrier-free construction and public misunderstandings of deaf people persist. These issues may relate to a lack of legal status and national standardization for CSL, combined with hearing-centered perspectives in the development of national policies regarding the deaf, which deserve deeper consideration in the improvement of Chinese barrier-free construction.

## Introduction

### Chinese Sign Language policies

Based on Stokoe’s (1966) study on American Sign Language (SL), SLs have been admitted as systemic languages parallel to spoken languages. This kind of language uses the visual-manual modality (e.g., facial expressions and body movements) to convey information and has its unique linguistic features (Lin et al., 2018). Since then, many countries (e.g., Sweden and Finland) have recognized their domestic SLs at the national level. Federations like the World Federation of the Deaf (WFD) have also called for attention to the rights of SLs and deaf people. Nevertheless, SLs in many countries nowadays are still seen as “inappropriate” in deaf education (Ladd, 2003), lacking standardization (Adam, 2015), considered malleable to change and replacement by manual codes (De Meulder et al., 2019), subordinated by spoken languages (Van Herreweghe et al., 2015), and even related to the disability of their users, the deaf people (Bauman & Murray, 2014).

Despite having the largest deaf population in the world (Xiaoyan & Ruiling, 2009), estimated at 20.57 million (The National Bureau of Statistics of China, 2025), China only recognized SL interpreting for deaf people as a profession in 2007. So far, only three regulations related to the promotion of SL have been published nationwide: The Action Plan for Standardization of Sign Language and Braille (2015–2020)^1^and (2021–2025)^2^, and The Notice on Accelerating the Promotion of the National Sign Language and Braille in Special Education School (2024)^3^. These policies only encouraged the usage and standardization of Chinese Sign Language (CSL) without recognizing its legal status specifically.

Some national deaf federations have been established to protect CSL and deaf people’s rights: the China Association of the Deaf (中国聋人协会, CAD) in 1956, evolving into the China Association for the Blind and Deaf (中国盲人聋人协会, CABD) in 1960; the China Disabled Persons’ Federation (中国残疾人联合会, CDPF) in 1988, and CABD became a subpart of it; the National Research Center for Sign Language and Braille (国家手语和盲文研究中心) in 2010, aiming to prepare for CSL’s standardization nationwide. However, these national organizations were not founded and run by deaf people, so deaf Chinese do not think these federations connect tightly with their communities (Lytle et al., 2005). So far, CSL is ignored by mainstream culture, and communicative barriers between deaf and hearing people still exist (Zheng & Zhao, 2020).

### Accessible retransmission of the 2025 China Media Group New Year’s Gala

The China Media Group (CMG) Chinese New Year’s Gala (Chunwan in Chinese) is a cultural and artistic evening gala produced by CMG every Chinese New Year’s Eve since 1983. It is a party celebrated by Chinese people worldwide, with the world’s largest audience for any TV show (Lim, 2012), estimated at over 16 billion for the 2025 Gala (CCTV News, 2025).

In January 2025, the National Notice on Further Strengthening the Public Cultural Services for People with Disabilities in Radio, Television and Audio-visual^4^ was published. Under this requirement, the 2025 CMG Chinese New Year’s Gala (referred to as “the Gala” thereafter) firstly provided “real-time accessible retransmission” (Disability in China, 2025) for the disabled, including SL Translation for the deaf and radio narration for the blind. This was also the first time that the SL Interpreters (SLI) of a Chinese TV program consisted mainly of deaf people, who were members of the China Disabled People’s Art Troupe. They creatively combined SL with aesthetic performances (e.g., dancing, drama) during the Gala.

Zheng Xuan, a deaf professor from Beijing Normal University, was invited to watch the SL Translation at the Gala’s rehearsal. Zheng suggested that “the comedies’ translation was natural CSL among the young signers. However, the generational differences in signing are significant, so the translation should be adjusted to ensure the senior signers can also understand the translation” (Disability in China, 2025). Apart from age, other factors, like education and ethnicity, also resulted in deaf people’s signing diversity (Hodge & Goswell, 2023). To make it understood by different signing audiences, Hodge & Goswell suggested that to create effective translations, clear lexicons known by most signers and integration of meaningful signing practices (e.g., interaction with spaces and contexts) should be used, in addition to natural SL.

Nevertheless, since this was the first time that SL Translation appeared in a nationwide popular TV program, with deaf people performing artistic SL to the Chinese public, Zheng noted that it is a chance for the public to understand SL as a language and art, which can be “cool and beautiful” (Disability in China, 2025).

### Research questions

Loh (2025a) contends that language ideology is reflected in both explicit comments about language and implicit meaning from daily discourses. To understand Chinese people’s language ideologies of CSL, this research took the Gala’s accessible retransmission as a case study, studying related online comments and posts from both hearing and deaf people. There are two main research questions. (1) How do people perceive the Gala’s SL Translation? (2) What are the reasons behind these perceptions? Specifically, what language ideologies (Uysal & Sah, 2024) are revealed from the reasons? Theoretically, the study contributes to the discussion of SLs and deaf people’s legal status in China. Empirically, the study interrogates the Chinese public’s understandings of SL and the voices of deaf people in China.

The paper begins with a literature review on CSL’s development (Section 2.1), the status of Chinese deaf people and CSL (Section 2.2), SL Translation in Chinese TV programs (Section 2.3), and language ideologies and analytical methods (Section 2.4). The corpus-building and qualitative analytical procedures are included in Section 3. The findings report on the quantitative study of public comments (Section 4.1) and the qualitative analysis of feedback from deaf people (Section 4.2). The implications are mentioned in Section 5.

## Literature

### Perception of Chinese Sign Language

Although it is generally believed in the research area that CSL generally refers to the Natural Sign Language (NSL) that has strong links to Chinese Deaf Culture and a different grammatical structure from spoken Chinese (Wang & Andrews, 2017; Zhao & Ye, 2019), its public perception can be complicated, since there has not been an official definition for it in national legislations.

The 2003 publication of 《中国手语》 (Chinese Sign Language), a later version of 《聋哑人通用手语草图》 (A Scheme of a General SL for the Deaf-Mutes) compiled by CABD, was the first to mention “Chinese Sign Language”. For a long time, people associated the concept with the book’s lexicons. Both versions above linked each sign to Chinese syllables or morphemes, creating the prototype of Signed Chinese (SC) with signed lexicons and Chinese grammar (Jones, 2013).

The implementation of these books failed (Gu et al., 2005; Jones et al., 2021). The National Research Center for Sign Language and Braille (2014) stated that 68% of deaf interviewees found the book “Chinese Sign Language” unhelpful. Reasons included: (1) its lexical items were invented and illogical, not aligning with deaf signing habits; (2) it lacks grammatical information, so hearing teachers mainly followed the grammar of spoken Chinese (Yuan, 2002); (3) deaf people preferred NSL, and rarely communicated with hearing people, making the lexicons in the book less useful (Yang, 2015). As a result, deaf Chinese preferred to define their own language as NSL, trying to distinguish it from the lexicons in the book.

In Mainland China, NSL can be divided into Northern (e.g., Beijing) and Southern (e.g., Shanghai) SL, mainly differing in lexicon and signing methods (Gong, 2009; Yang, 2015). Usually, these NSLs are mutually intelligible, and mutual influences among those varieties (Yang, 2008) are common in deaf communities and schools. However, exceptions exist because of ethnic or regional effects. China is a country with a large area, various cultures, and the largest number of deaf people in the world, and it failed to provide convenient conditions for communication in the past, resulting in a great variety of NSLs from different places, especially those that were far away from the mainland (Zheng & Zhao, 2020). The Tibetan SL, officially recognized as a minority SL in China since 2000, is significantly different from the Southern and Northern SLs (Hofer, 2020; Yang, 2015). The Taiwanese, Hong Kong, and Macau SLs differ from their Mainland counterparts as well (refer to Wang & Li, 2015 & Yi, 2022 for more information). This not only strengthened the difficulties of CSL standardization but also affected the public understanding of CSL.

In 2018, the book “Chinese Sign Language” was revised by the Center for Special Education of Beijing Normal University and renamed as《国家通用手语词典》 (National General Sign Language Lexicon). The Lexicon collected the simplest and most practical signs among different regional NSLs across China. Public perception of this new version is yet to be studied. Yet the continuous revision and reapplication of referential materials for a national SL have led to a noticeable gap in SLs between younger and older deaf signers (Yang, 2015), as well as deeper confusion of public perception on CSL.

Additionally, due to the lack of standardization, teaching strategies are multimodal in deaf schools, mainly consisting of four types of signing practices (see Table 1). SC and Pinyin Finger Spelling are supplements to oral communication, and SC did not have NSL’s linguistic features (e.g., facial expression, verb agreement) and followed the grammar of spoken Chinese. Chinese character signs are signs imitating or air-writing Chinese characters (Luo, 2008; Wang & Andrews, 2017). SC, which is less clear and practical for deaf people, was regarded as the “standard” signing in national policies due to the dominance of spoken Chinese. In this way, the public perception of CSL became more complicated.

**Table 1:** Types of signing practices in Chinese deaf education.

Ways	Features
Natural Sign Language	Spontaneous hand gestures used and developed by deaf people
Signed Chinese	Signed lexicons (natural or invented) with the grammar of spoken Chinese
Chinese Character Signs	Imitating the form of the Chinese characters or writing them in the air
Pinyin Finger Spelling	Fingerspelling the phonetic alphabet of the lexicon’s pinyin

As a result, most Chinese people cannot distinguish the differences among CSL, SC, Fingerspelling, Chinese character signs, and even body language. To deal with this situation, scholars have started to study NSL’s linguistic features since 1979, trying to promote CSL’s standardization and public perception (Yu & He, 2009). So far, scholars have researched its lexicon (e.g., Fischer & Gong, 2010; Gong, 2005; Gu, 2005), grammar (e.g., Sun, 2003; Wu & Li, 2012), and developed a corpus of CSL (Zhao et al., 2017).

However, as mentioned in Section 1, national policies on CSL promotion have been limited, and those policies only applied the term “国家手语” (National Sign Language) instead of “中国手语” (CSL), without giving any specific definition of both terms at the national level. And all those policies have not been conducted well, since there is no “standard” of CSL and not enough people with SL competencies. Some non-governmental deaf federations and regional organizations have recently been working to promote the “National General Sign Language” by hosting SL parties and lectures (e.g., Sign Language Island in Zhejiang Province, Sound Giving in Chongqing, and others), but their influence has not spread nationwide and is mostly concentrated in cities with a strong Deaf Culture presence (e.g., Shanghai, Hong Kong, Zhejiang, and Chongqing). To enhance public understanding of CSL, greater efforts are needed at the national level.

### Status of Chinese Sign Language and deaf Chinese

According to WFD’s official statistics in 2025, China was not included in the 81 nations with legal recognition of their domestic SLs^5^. Until 2021, among the 90 national laws and 50 regulations about the disabled in China, there were only lines about promotion of National Sign Language, but no special clause defining CSL’s legal status (Feng, 2021; Jiang & Yang, 2018), as what has mentioned in Sections 1.1 &2.1.

Both hearing and deaf Chinese agreed that CSL standardization is a requisite for its legal recognition (Jones et al., 2021), due to the large regional varieties of Chinese NSL (Zheng & Zhao, 2020). However, SC still dominates the process of standardization, and NSL is not regarded as a linguistic right, but a communicative option (Yang, 2011 & 2015). Over the decades, both deaf individuals and federations have been trying to signify the importance of NSL (e.g., Yau, 1977a; Yau, 1977b; Dai, 1996; Yang, 2015), both in culture and everyday life.

Historically, deafness was labelled as a “mental disorder” and one of the four evils (四奸) of personal qualities: Deafness, Ignorance, Obstinacy, and Arrogance (“聋、 昧 、 顽 、 嚣,” Zheng, 2022). Therefore, both families and schools with deaf children regarded voicing the dominant language as a measure of the children’s success (Lytle et al., 2005; Xiaoyan & Ruiling, 2009), and neglected the significance of CSL, impeding its popularity and benefits for supporting deaf children’s communication development.

Due to the cultural tradition of hiding family shortcomings (家丑不可外扬), Chinese families always limit their deaf children’s socialization and suppress the children’s facial expressions or extravagant body movements in public. Consequently, deaf Chinese have suffered from social isolation and discrimination (Shen, 2013).

In schools, the Oral/Aural (O-A) teaching approach emphasizes deaf children’s speech and listening skills development. SC and fingerspelling, instead of NSL, took the role of teaching supplements. In other words, the role of NSL was neglected (Wang & Andrews, 2017; Yang, 2015). However, only children who were not born with complete deafness tended to succeed under this approach, and most deaf children found it difficult or impossible to follow the curriculum (Lytle et al., 2005). The approach’s failure led to curriculum change with lower expectations (e.g., mastering fewer Chinese characters) in 2016, which, in turn, underestimates deaf people’s cognitive and language abilities and enforces low expectations for deaf people’s educational and social outcomes (Wang & Andrews, 2017).

Teachers of deaf children are not required to learn CSL (Zheng & Zhao, 2020), and the number of deaf teachers, who typically lack speech privileges, declined significantly after the increasingly stringent requirement for all teachers to speak fluent Chinese (Wang & Andrews, 2017). Consequently, SLI Services (SLIS) are limited in schools (Jones et al., 2021). This situation leads to “language deprivation” (Gulati, 2018) of deaf children, who lose chances to fully develop their first language (i.e., CSL) due to insufficient exposure to linguistic input in their earliest childhood.

Many deaf children thus have limited signing abilities and rely on writing or speaking with a “deaf accent (i.e., dysfluency and non-standard pronunciation due to hearing loss)” (De Meulder et al., 2019), complicating the possibility of CSL standardization and effectiveness of SLIS (Hodge & Goswell, 2023).

Additionally, the “accessibility” of SLIS has been questioned by deaf Chinese. SLIs mainly work part-time and are not trained beforehand, so SLIS remain largely incomprehensible to deaf audiences. Over half of the SLI interviewed by Xiaoyan & Ruiling (2009) applied oral language at 60%–80% in their interpretation, which deaf people found difficult to understand. When communicating with hearing people, deaf people prefer writing, lip reading, or hearing aids, instead of SLIS. The “inaccessible” SLIS contributes to and even exacerbates the “Illusion of Inclusion” in society, by perceiving it as a supporting access without considering its quality and usability (Caselli et al., 2020; De Meulder & Haualand, 2021). Under this illusion, the administrators and the public assume that deaf people can conveniently participate in social activities like education, public services, and so on. Therefore, no other barrier-free constructions are provided, nor further improvements are made. Consequently, the actual inaccessibility of SLIS creates larger language barriers and reinforces deaf people’s unequal social status and their sense of “distantism,” i.e., a feeling of standing apart due to the public’s privileging of hearing and vision (Clark, 2025).

Since deafness is usually unrecognizable through appearance, deaf people are more easily neglected across all areas of society than other disabled people. Zimu (2020) shared that there is less visual equipment in public services than audio equipment or blind trails for blind people, and that when the government asked for feedback from the disabled about the “accessible construction” services, the task was completed through telephoning, which is difficult and even impossible for deaf people to access.

The public often misconstrues deaf people as impolite when they do not reply instantly or even ignore the conversation because they did not hear. Consequently, people tend to lose patience if communication fails, causing “secondary disability” in socialization beyond their “first disability” of hearing loss. Supporting services like cochlear implants or hearing aids also incur extra costs, and some deaf individuals must seek permission to use them at work and school (Dongdong, 2020).

### Sign Language Translation on Chinese TV programs

Currently, 28 of 30 provincial TV channels and 142 of 6,557 municipal652955 channels in China provide SL Translation weekly or daily (Xiao et al., 2020; Xiao & Li, 2018), making China one of the countries with the most TV programs using SL Translation (Shi, 2018).

However, generally, the SL Translation provided on Chinese TV channels greatly disappoints the audience. Firstly, because there were fewer people with SL competence and hearing people tend to communicate better with other staff during the TV programs, the SLIs in these programs were often hearing staff from deaf schools or communities, who had not been well-trained or qualified beforehand. They happened to learn SL when they were working with deaf people or growing up in a deaf community, so they signed mainly with a mix of SC, NSL, and fingerspelling. This deviation from NSL reduces comprehension, affecting the quality and fidelity of the information from the interpretation (Jiang, 2004; Xiaoyan & Ruiling, 2009; Xiao et al., 2020). Xiaoyan and Ruiling’s (2009) survey found that most SLI felt they should be loyal to their employer, signing in a “standard” way regardless of deaf viewers’ understanding. But what is “standard” when CSL lacks legal standards (Clark, 2014) and deaf signs are diverse (Hodge & Goswell, 2023)? In 2009, the National Occupational Standards for SLI were published, focusing only on lexicon and sentence translation, ignoring practical communication (Xiao et al., 2020). As a result, SL Translation on Chinese TV is criticized as an “Image Project,” prioritizing national image over deaf audiences’ needs (Li, 2013).

Wang and Li (2015) found that deaf TV viewers in Mainland China preferred deaf hosts from Taiwan to hearing ones from Mainland China, even though they cannot fully understand Taiwanese SL. This is because the deaf hosts use natural facial expressions, language fluency, and beautiful gestures, which all aid clarity and comprehension. Wang & Li concluded it as “自己人效应” (The Phenomenon of Preferring People on One’s Own Side).

Secondly, SL Translation was mainly provided in news about the hearing society, with less relation to deaf communities, which deaf viewers found boring (Kyle, 2007; Zeng, 2018).

Thirdly, the translation was usually added after the recording of the spoken news, so signers don’t receive the news at the same time as hearing people (Gao & Liu, 2015).

Finally, the unnaturally fast signing to match the speaking speed and the small screen for translation also affected deaf people’s experience of watching those TV programs (Wang & Li, 2015).

Recently, China has been continuously trying to find suitable ways for SL Translation on TV, such as applying an AI interpreter (Xiangli, 2023) and testing new interpreting modes (e.g., signing while speaking, which adapts speech to signing, Liu, 2020; Yang, 2022). The Gala’s SL Translation is a part of the attempt. By analyzing the online comments on the SL Translation, this research may contribute to the improvement of Chinese TV programs’ SL Translation.

### Language ideologies, corpus-assisted discourse studies, and qualitative review

After Silverstein (1979) applied the concept of “Linguistic Ideology” (equals to “language ideology,” Loh, 2025a: 6), the study of language ideologies has been meaningful for sociolinguistics and linguistic anthropology (Irvine, 2012; Kroskrity, 2004). Irvine (1989: 255) concluded language ideology as a “cultural system” of thoughts about the relationships between society and language, and the “moral and political interest” behind them. Studying the general language ideology of a language can confer the cultural and political ideology about the language and its users. As Terje Basilie, a Norwegian psychologist, has said, accepting a person means you have accepted their language, so if you reject their language, you also reject the person who uses it (cited from Shen, 2013). As the public attitude towards a language is always recontextualized by their group leader (Myers-Scotton, 2006), investigation of language ideologies can benefit from public discourses both explicitly and implicitly (see Section 1).

Although the study of language ideologies has been ongoing for many years, exploring ideologies in SL and deaf studies is a relatively new field (Kunsters et al., 2020). Kunsters and colleagues assert that language ideologies are closely connected to language practices, which change “across space and moments in time” (2020:3). Therefore, SL ideologies should be analyzed in a specific situation and environment, and can be understood through practices such as its naming, standardization, classification, and public perceptions.

For example, Haualand and Holmström (2019) studied the attitudes toward signers by assessing the national statuses of Norwegian and Swedish SLs, and found the significant role of national language policies in public perception of SL and its users; while Loh (2025a) described deaf and hearing people’s ideologies from their competing discourses about Jordanian SL (LIU), and revealed that both the positive and negative description of LIU are central to Jordanian deaf people’s self-identities. These studies revealed the role of discourses in ideological analysis and the interaction between national policies and individual understandings of language.

Corpus-assisted discourse study (CADS) helps investigate language ideologies, which grasp the recurring items (i.e., themes) from the discourse data to reveal its central information (Baker et al., 2008). Also, focusing on specific texts with core linguistic patterns (e.g., keywords and lexical bundles) allows the researcher to discover the implicit linguistic phenomena not easily observed by purely quantitative or qualitative studies (Baker et al., 2013). For example, Krendel et al. (2022) studied social actors’ gendered representation in manosphere communities with CADS based on keywords, and Hansson and Page (2022) studied the discourses of 19 UK politicians based on their recurring lexical bundles to conclude their strategies of blame avoidance. These studies gained fruitful information that would not otherwise have been identified.

In the humanities and social sciences, it is common to focus on specific texts qualitatively for deeper understanding after quantitative measures of keywords, collocations, and so on (e.g., Kopf, 2020; Ross & Rivers, 2020). In the process of qualitative analysis, Jones et al. (2021) signified the “focus group discussion”, i.e., analyzing the statements and themes through the lens of participants in the events (Corbin & Strauss, 2014), for revealing the actual effectiveness of the events for these participants.

To gain a comprehensive understanding of Chinese public’s views on CSL and deaf people, the current research was divided into two studies: (1) a CADS with keywords and collocates based on thematic analysis of comments from RedNote (a social media platform in China), to figure out the public perception of CSL among people who watched the Gala, and (2) a qualitative analysis of nine RedNote/Bilibili (Chinese social media platforms) posts from deaf Chinese who watched the Gala, to understand the needs of deaf Chinese specifically, as they are the target beneficiary of SL Translation.

## Materials and Methods

### Study 1 Corpus-assisted Discourse Studies with a thematic approach

#### Materials

**Figure 1:**
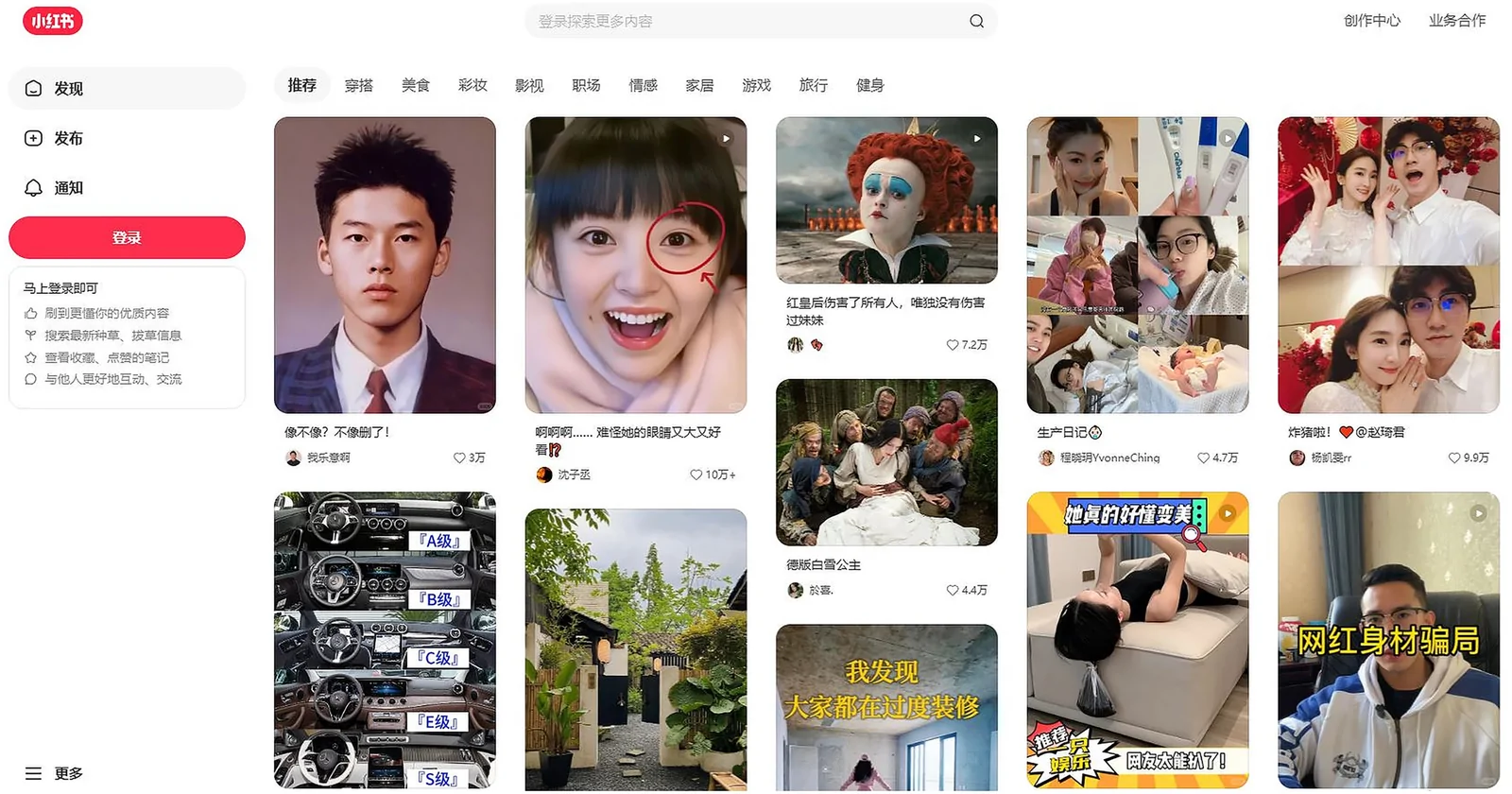
Screenshot of the RedNote homepage.

The social media platform RedNote (Xiaohongshu in Chinese, Figure 1) initially served as a fashion guidance platform for Chinese users and has recently evolved into an information and life-sharing app (Ipsos, 2020). As a new social media community, its user base has grown steadily day by day. By mid-2025, its monthly active users reached 0.35 billion, surpassing other Chinese social media platforms significantly (Wang, 2025). People from various backgrounds are attracted by its unique community features that encourage sharing and commenting on daily lives. To better understand the general perception towards CSL, and because ideologies can change over time (Kunsters et al., 2020), more recent data are preferable. Therefore, RedNote was selected as the primary source of corpus data.

#### Methods

Study 1 builds a corpus of comments on the SL Translation in the Gala and divides them into various themes to understand the primary ideas in public in advance. Then, unique keywords and collocations are analyzed under different themes by comparing them with a reference corpus consisting of randomized texts about deaf people and SL, to show people’s perception in detail.

Several stages are involved in corpus building. Firstly, the keywords “春晚无障碍转播” (the Gala’s accessible retransmission) and “春晚手语” (the Gala’s SL) were searched on RedNote to locate the thematically linked posts and comments on them. To better focus on the public’s perception of the interpretation, posts with more than 30 comments were included in the research, and 25 posts (Appendix A) met this criterion.

**Figure 2:**
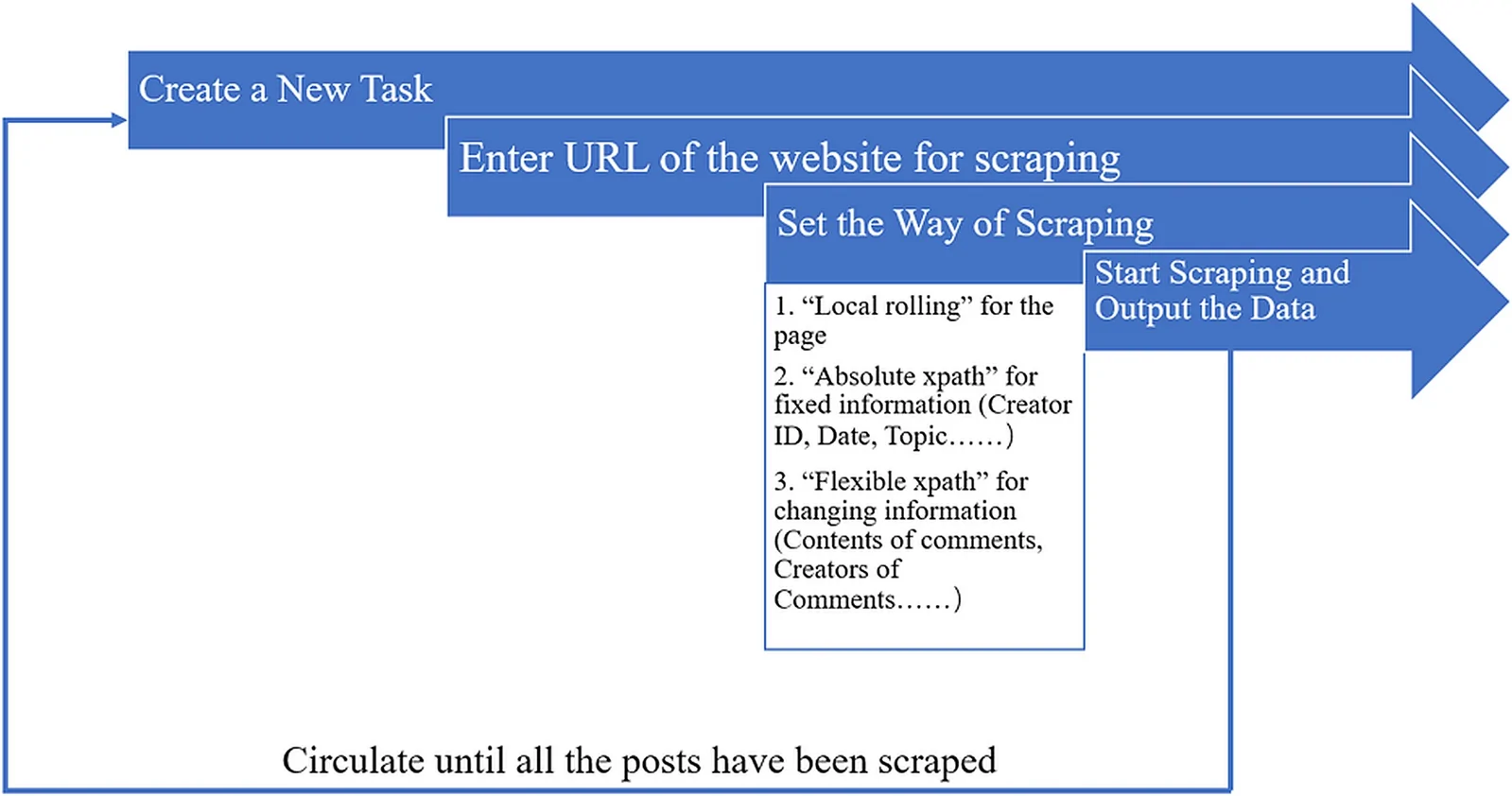
Process of scraping with Octoparse.

A total of 6,538 RedNote comments from these 25 posts were scraped by Octoparse V8.4 (Octopus Data Inc., 2023). This is a web scraper developed by a Chinese software company, Octopus Data Inc., so it is useful for scraping Chinese texts. Also, the application is coding-free (cf. Python), so the scraping process is easy (Figure 2). After scraping, unrelated comments, such as those mentioning other users, New Year’s wishes, evaluation of the original performance (i.e., evaluating the performers instead of the SL Translation or the SLI), and emojis without extra meaning (i.e., emojis that only strengthened the meaning of the texts), were excluded. This left 2,424 comments for analysis here.

**Figure 3:**
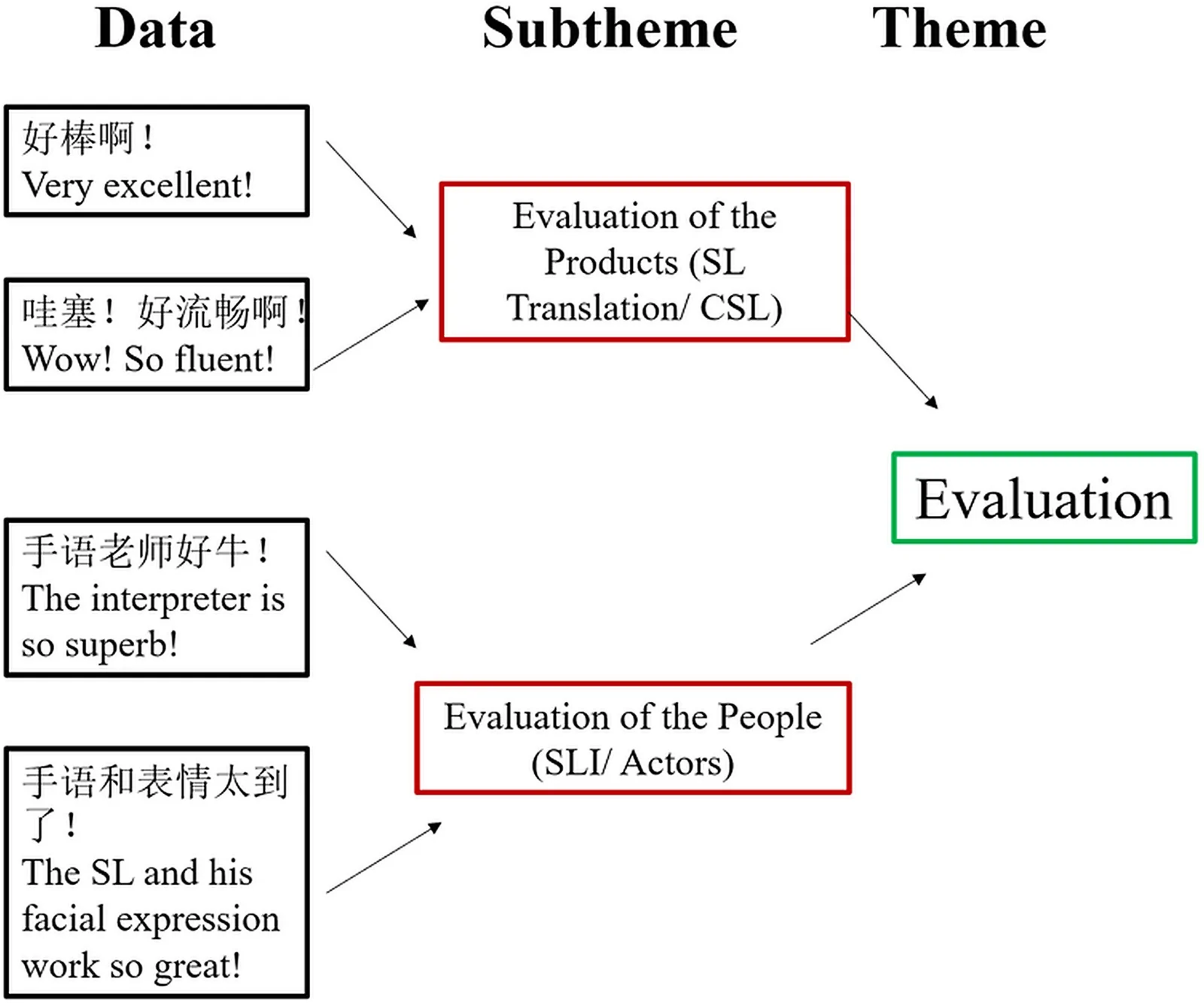
Example of thematic coding from gala comments on RedNote.

Previous studies of language ideologies reflected in social media discourses (e.g., Shepherd et al., 2015; Wang, 2023) have qualitatively analyzed the thematic threads of data. To explore their repeated ideas, this research coded the comments thematically in Excel with a similarly inductive approach (i.e., developing codes from the data itself, rather than from the existing theories). Figure 3 shows an example of coding.

As the boundaries between characters and words are not clearly defined in Chinese (Huang & Xue, 2015), and Octoparse only scrapes texts without parsing, it is challenging for corpus tools to recognize words in Chinese texts. Therefore, before analysis, the scraped texts were further parsed by a Chinese parsing tool, the Corpus Word Parser (Institute of Applied Linguistics of the Ministry of Education, 2014). Afterwards, keywords and collocates were identified based on various coded themes, such as “Evaluation,” and subthemes, such as the evaluation of the products (SL Translation/CSL) and the people performing in the Gala (SLI/Actors) showing in the example above.

Since language ideologies are flexible “across space and moments in time” (Kunsters et al.), to better understand people’s perception of SL Translation in the Gala, collocates and keywords for each theme were then calculated by AntConc 3.5.9 (Anthony, 2021) with a specialized reference corpus (30,000 samples) built with randomized texts downloaded from the multi-field corpus produced by the BLCU Corpus Center (Xun et al., 2016).^6^ This specialized reference corpus contains Chinese newspapers, literature, and social media, with specific searched words “聋人” (signing deaf), “手语” (SL), and “听障” (speaking deaf), which all relate to the topic of this study. Keywords in the target corpus were then ranked by log-likelihood (shown as “Keyness” in AntConc) to explore their uniqueness, showing the corpus’s unusual linguistic features (Scott, 1997). Keywords with log-likelihood over zero are those unique in the target corpus, while those with log-likelihood under zero are shared in both corpora (Baker & Vessey, 2018). Study 1 focused mainly on the keywords with log-likelihood over zero, which showed the specific contents of the public perception of CSL and deaf people after the Gala’s SL Translation.

#### Study 2 qualitative review: Emic perspective from deaf Chinese viewers

##### Materials

“Nothing about us without us,” the tenet of the disability empowerment movement (Jones et al., 2021), emphasizes the importance of involving “the researched” and “the focused” (i.e., the disabled) in research. Similarly, there should be no discussion about deaf people without including their voices, so the perspectives of Chinese deaf individuals on the SLT in the Gala should be taken seriously.

Due to the issues of deaf education (see Section 2), many deaf people have limited ability to leave comments on social media, so their voices are not included in Study 1. Also, the population of deaf people is smaller than hearing people, so the corpus data in Study 1 mainly includes hearing people’s views and opinions. Therefore, it is necessary to supplement with Study 2, which focuses on feedback from deaf Chinese.

**Figure 4:**
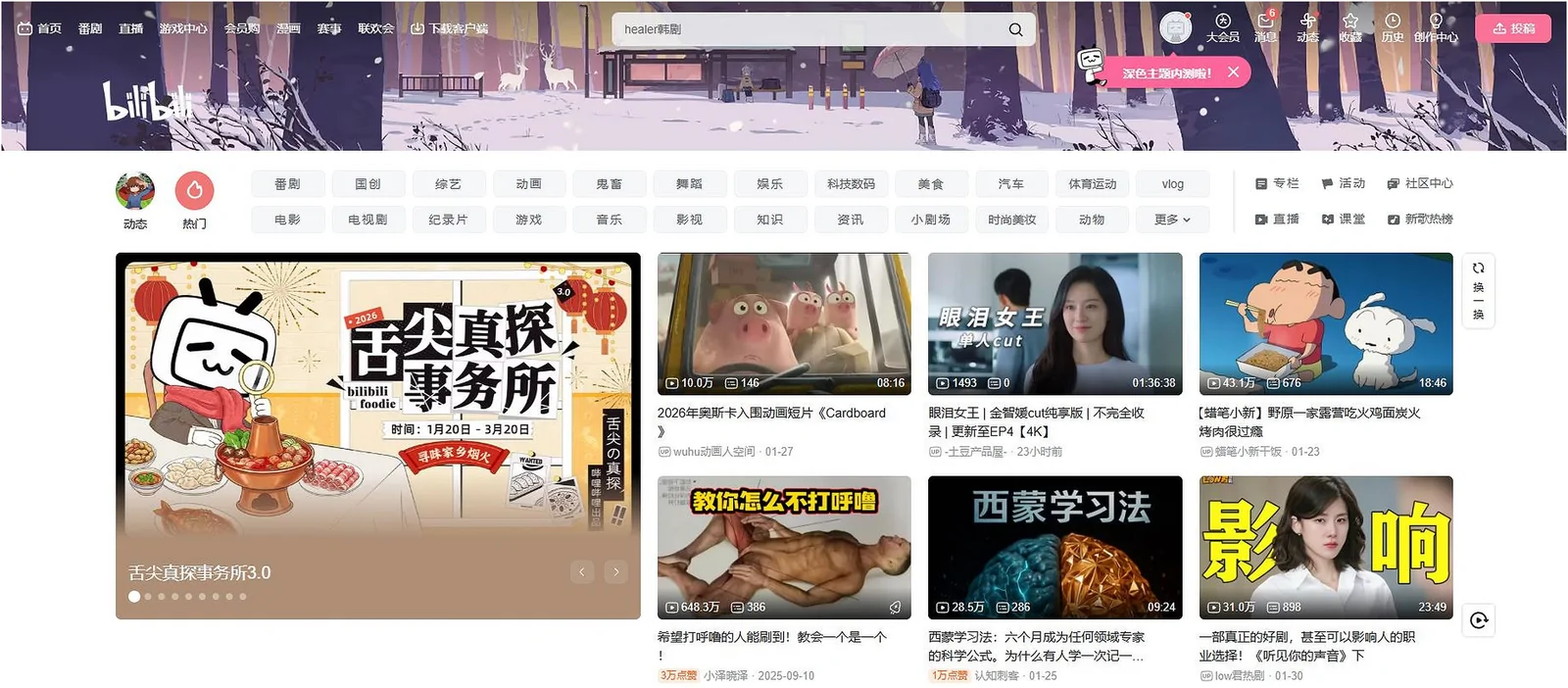
Screenshot of the Bilibili homepage.

To examine deaf people’s perception of the Gala’s SL Translation and its underlying ideologies, this study searched the 2025 top five popular Chinese social media platforms ranked by QuestMobile, a social media detecting platform in China^7^ (RedNote, TikTok, Bilibili, Kuaishou, and Wechat Channels), using keywords “春晚无障碍转播” (the Gala’s accessible retransmission), “聋人” (deaf people who mainly sign), and “听障” (deaf people who mainly speak). Data selection was guided by the PICO (Population-Interventions-Comparators-Outcomes) framework (Lockwood et al., 2015; Murdoch University, 2025), focusing on research questions, context, and target population. Criteria included: posts by deaf Chinese, relevance to the Gala’s SL Translation, and feedback on the SL Translation. Eight posts from RedNote and Bilibili met these criteria, plus one from a blind RedNote user reflecting common issues, which was included (Appendix B). “大巧子” uploaded two posts before and after the Gala, they are useful for comparing her views. Bilibili is a Chinese video-sharing website where users can upload their original or re-edited videos, similar to YouTube (Figure 4).

Table 2 shows that the posts are various in language forms, from spoken Chinese to CSL. Therefore, these reviews can represent the opinions of deaf people with different language preferences and thus contribute to a more comprehensive picture of the Gala’s SL Translation.

**Table 2:** User information of the analyzed posts.

Name of user	Deaf/blind	Language form used in the post
DUDU 杜银铃	Deaf	Spoken Chinese, CSL, Subtitle
大巧子	Deaf	Spoken Chinese, CSL, Subtitle
栾晓煜	Deaf	Spoken Chinese, Subtitle
太棒了伞	Deaf	CSL, Voice-over, Subtitle
有风吹过你的气息	Deaf	Written Chinese
长颈鹿张涛	Deaf	CSL, Voice-over, Subtitle
智镜创研	Deaf	CSL, Voice-over, Subtitle
晴晴本情	Blind	Spoken Chinese, Subtitle

#### Methods

As all the posts contain subtitles, the transcription was based on the subtitles and adjusted according to the spoken Chinese or voice-over of the video. The CSL data cannot be analyzed here because the researcher does not know CSL. The edited transcripts were coded in NVivo 12.0 (Lumivero, 2017), an application for qualitative coding and information visualization.

**Figure 5:**
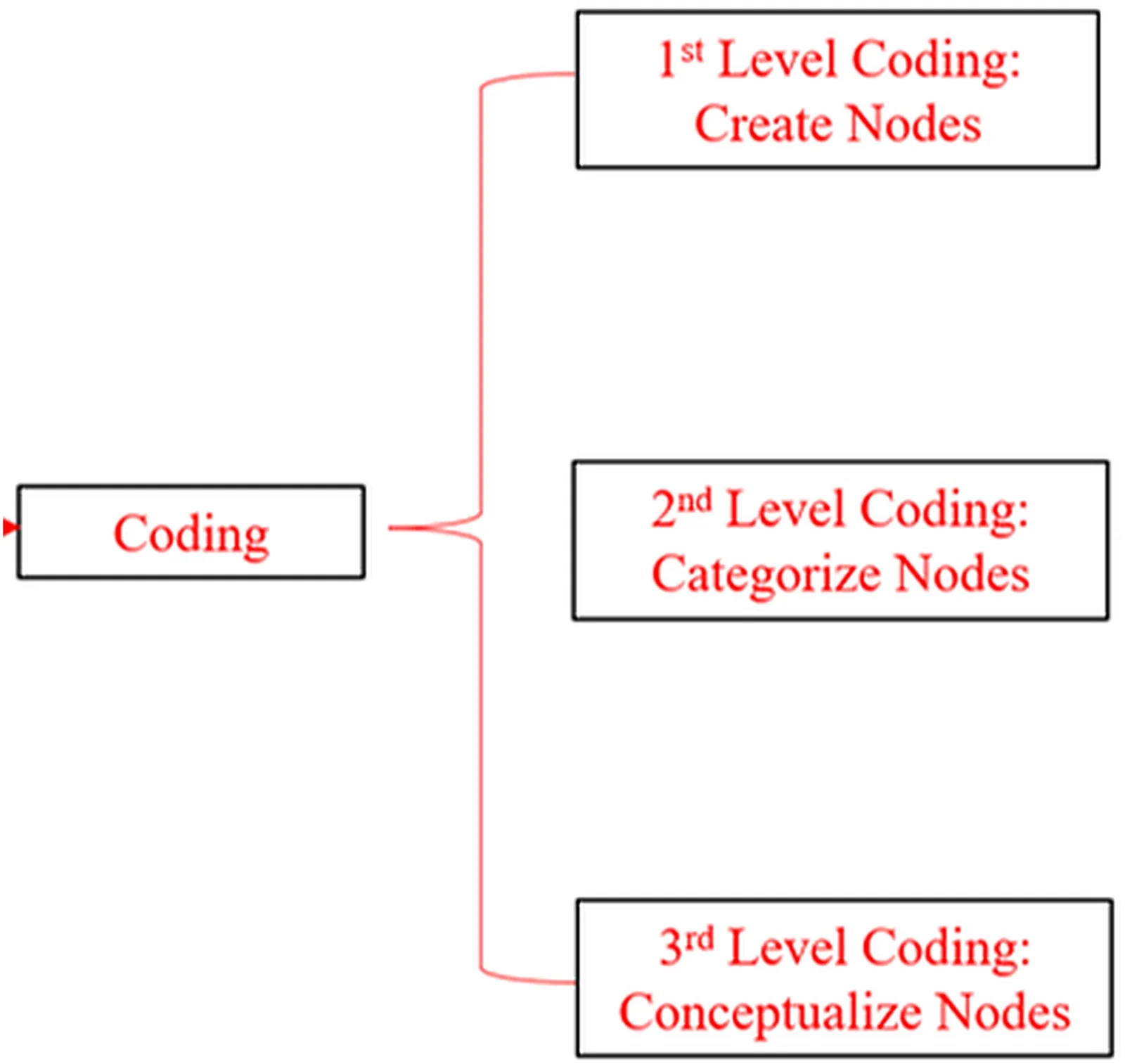
Process of coding in NVivo 12.0.

**Figure 6:**
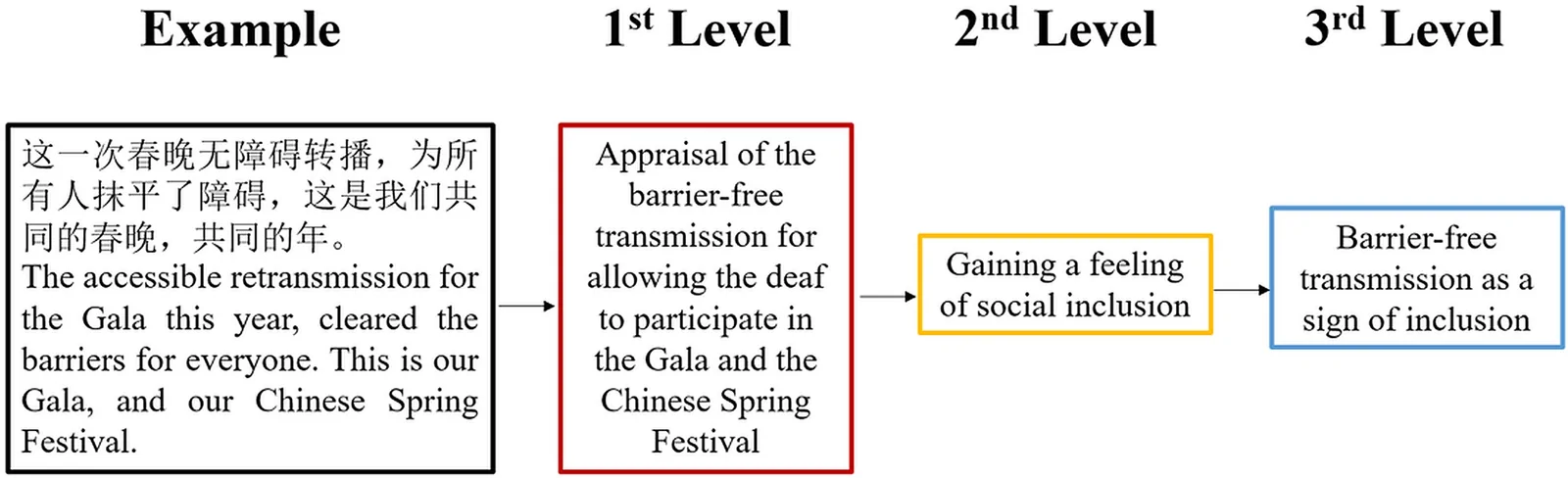
Example of the three-level coding of the online reviews from deaf Chinese.

In the operating process of NVivo 12.0, coding is the central part (Figure 5), which consists of three levels: The 1^st^ level is to create nodes while reading or rereading the transcripts; the 2^nd^ level is to review the nodes and merge similar items into one node or delete the unnecessary nodes; and the 3^rd^ level is to abstract the recurring themes among the nodes. An example of coding is provided in Figure 6. In this study, the users’ accounts of their attitudes and opinions are prioritized in the coding (Braun & Clarke, 2006). The following analyses are based on coded themes.

## Results

### Study 1

**Figure 7:**
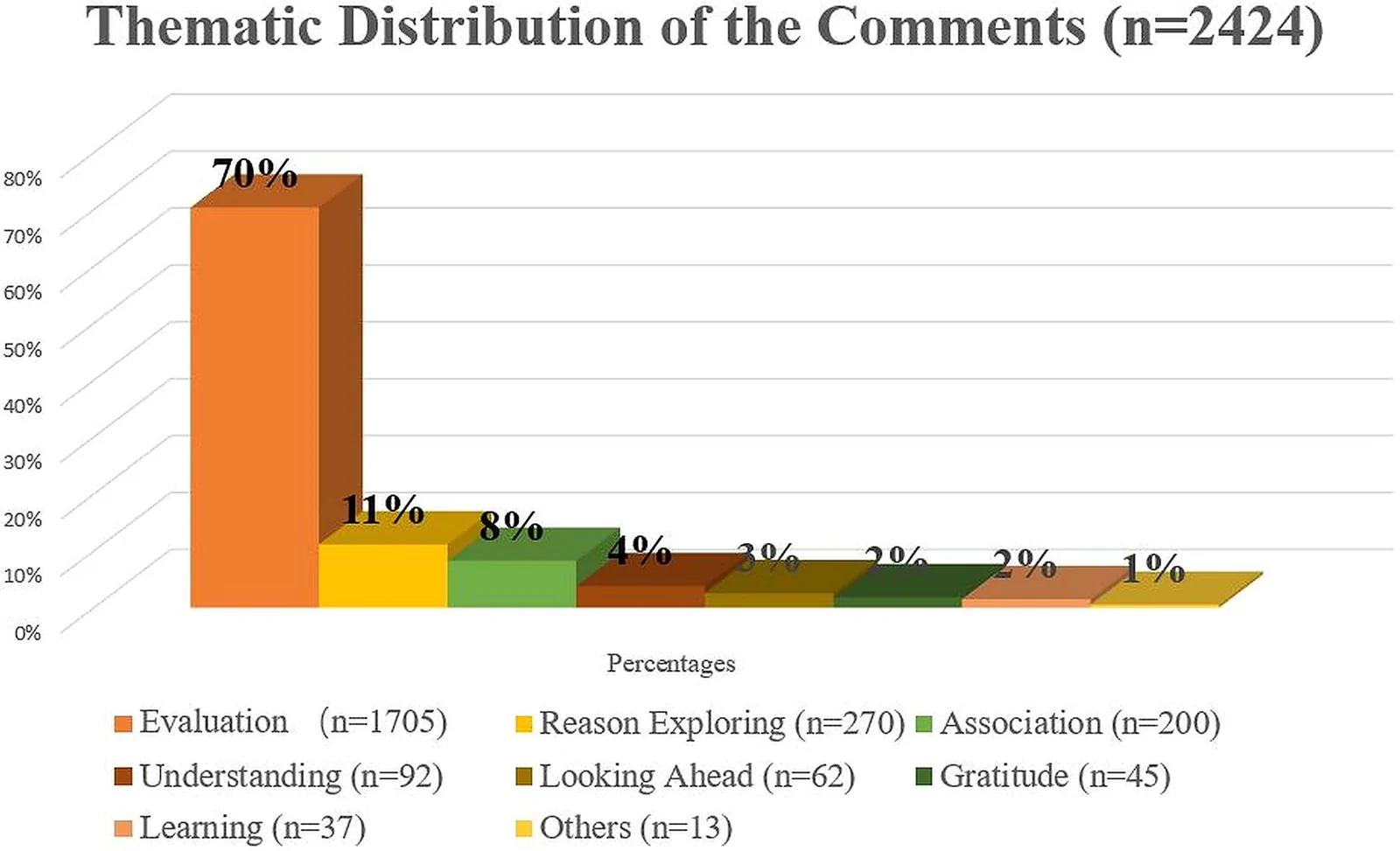
Thematic distribution of the comments on the SL translation in the gala (*n* = 2,424).

Based on qualitative coding of data from the Gala, eight recurring themes of discussion were identified within the comments (*n* = 2,424, see Figure 7). These themes were coded twice with a one-month interval to ensure the coding’s reliability. The full list of coded comments is available in the following Open Science Framework repository: https://osf.io/y6nbc/.

Each theme’s subtheme can be found in Table 3. The three primary themes: Evaluation (*n* = 1705; 70.34%), Reason Exploring (*n* = 270; 11.14%), and Association (*n* = 200; 8.25%) will be analyzed in detail in the following sections. This is because these three themes consist of the main part of the comments and are closely related to the research questions. The other five themes are saved for future studies.

**Table 3:** Thematic details of the comments (*n* = 2,424).

Themes	Number	Subthemes	Number
Evaluation	1705	Evaluation of the Products (SL Translation/CSL)	1,264
		Evaluation of the People (SLI/Actors)	441
Reason Exploring	270	Providing a Potential Reason for Offering SL Translation	142
		Questioning the Necessity of SL Translation	128
Association	200	SL or Deaf People-Related Knowledge Sharing	156
		Personal Experiences with SL or Deaf People	37
		Previous Deaf People Performance or SL Translation Sharing	7
Understanding	92	Showing Understanding to Deaf People	55
		Suggestion for Watching SL Translation without Sound	37
Looking Ahead	62	Suggestions for Future Facilitate	48
		Expressing Best Wishes to Barrier-free Construction	14
Gratitude	45	Appreciation for the Country	25
		Showing Gratitude to the Interpreters and Other Related Staff	20
Learning	37	Showing Gained Knowledge Related to SL or Deaf People	28
		Expressing Enthusiasm for SL Learning	9
Others	13	Comments not tightly related to the themes above	13
Total	2,424		

#### Evaluation

The “Evaluation” theme (*n* = 1705) includes comments that evaluated the SL products (*n* = 1,264) and the SLI (*n* = 441). To understand the evaluative meaning within the texts, sentiment analysis (Taboada, 2016) was applied to this data. According to Taboada, sentiment, i.e., the expression of subjectivity (positive, negative, or neutral) about something, can be analyzed from the texts in a language. Applying the Sentiment Lexicon (27,466 words in total) developed by the Dalian University of Technology Information Retrieval Laboratory, the keywords of the evaluative comments were categorized into various sentiments.

The Sentiment Lexicon categorized words into three sentimental polarity levels: 0 for neutral, + for positive, and – for negative. Polarity intensity ranges from 1, 3, 5, to 9, indicating low to high strength. For instance, “好” (good) is marked + with an intensity of 5, signifying it as a word with moderate positive emotion.

**Figure 8:**
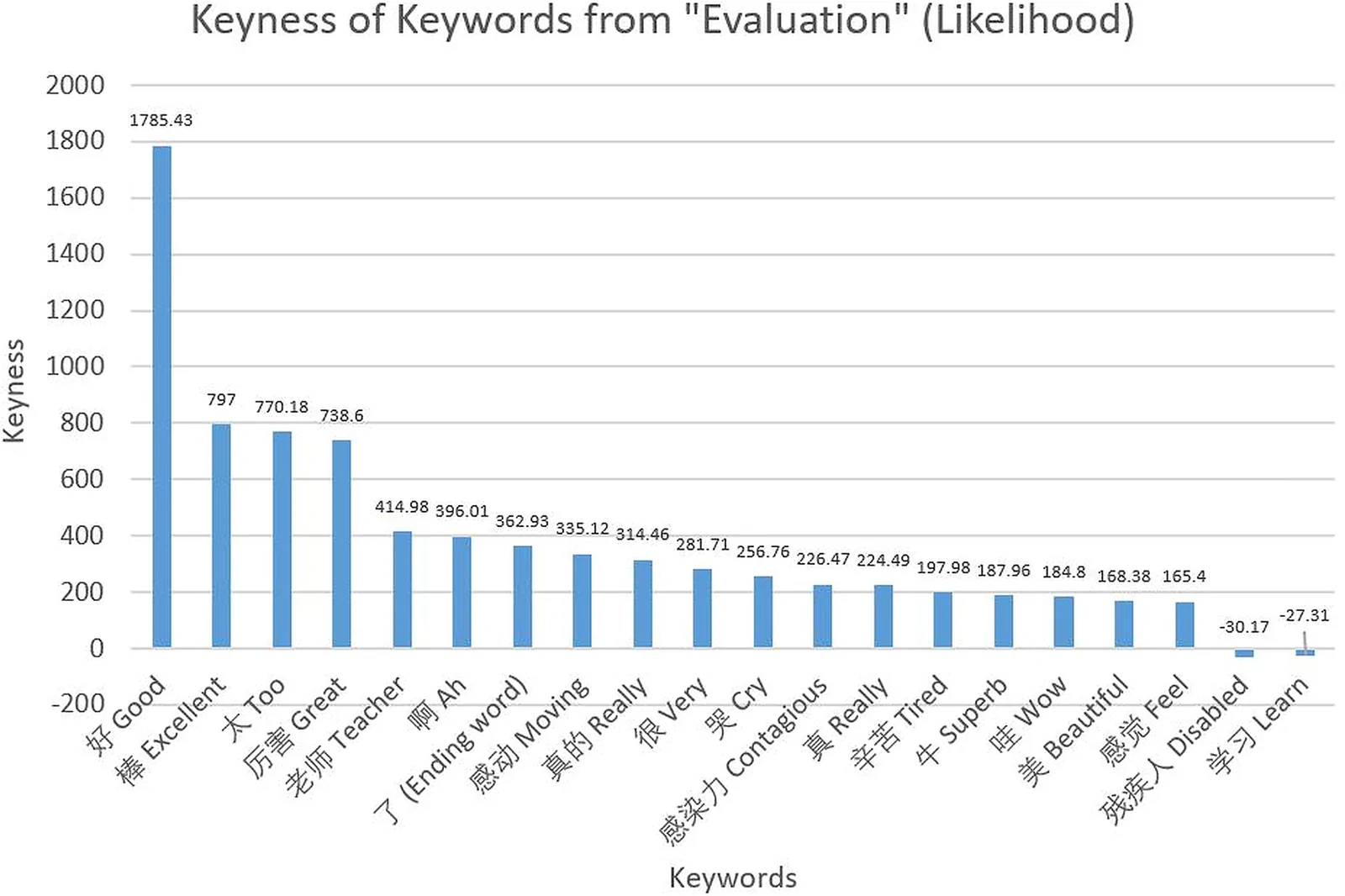
Keyness of keywords in comments of “Evaluation” theme.

Due to the large number of keywords with log-likelihood over zero, only those whose log-likelihood was over 150 were included to focus on the main views (Figure 8). Among the keywords, “好” (good), “棒” (excellent), “厉害” (great), “老师” (teacher), “感动” (moving), “哭” (cry), “感染力” (contagious), “牛” (superb), “哇” (wow), and “美” (beautiful), “残疾人” (disabled), and “学习” (learn) are sentimental lexicons that can be identified into various sentimental categories in the Sentiment Lexicon (Table 4).

**Table 4:** Sentiment analysis of the keywords in comments of “Evaluation” theme.

Keyword	Polarity	Intensity
好 Good	+	5
棒 Excellent	+	5
厉害 Great	+	9
老师 Teacher	+	5
感动 Moving	+	3
哭 Cry	0	5
感染力 Contagious	+	5
牛 Superb	+	7
哇 Wow	+	7
美 Beautiful	+	5
残疾人 Disabled	−	7
学习 Learn	0	5

Polarity: + = Positive; 0 = Neutral; − = Negative

Intensity: rank with 1, 3, 5, 7, and 9 (1 means the least intense, while 9 means the most)

According to Gee (2016), a word’s meaning can be changed by the situation of usage. Therefore, for the non-sentimental keywords like “太” (too), “啊” (ah), “了” (ending word), “真”/“真的” (really), “很” (very), and “感觉” (feel), which can modify both negative and positive words, their collocation patterns were analyzed and ranked by frequency (Table 5).

**Table 5:** Collocation patterns of the non-sentimental keywords.

Keyword	Collocate	Frequency
太 Too	棒 Excellent	64
	厉害 Great	51
	好 Good	22
	牛 Superb	21
	有感染力 Contagious	17
啊 Ah	棒 Excellent	29
	厉害 Great	18
	牛 Superb	10
了 Ending Word	哭 Cry	44
	棒 Excellent	40
	厉害 Great	38
	牛 Superb	15
	有感染力 Contagious	13
真的/真 Really	好 Good	67
	很 Very	24
	棒 Excellent	10
很 Very	好 Good	17
	棒 Excellent	15
感觉 Feel	好 Good	11

Sentiment analysis of the collocations refers to Table 3.

Based on Tables 4 and 5, the data in the target corpus show positive semantic prosody (Shahzadi et al., 2023), with most texts conveying an intense emotion. The structures “太 adj.了” (too adj.), “真的很  adj.” (really very adj.), and “adj. 啊” (similar to the usage of the exclamation “!”) were frequently used to emphasize emotional meaning, and the adjectives inserted into those structures were intense positive words (see Examples 1, 2, and 3). Compared with the common keywords in both corpora, “残疾人” (disabled, negative) and “学习” (learn, neutral), the unique keywords in the target corpus show that people significantly hold positive attitudes toward CSL and SLI in the Gala.

**Example 1**

“太 adj.了” (too adj.)

他们太厉害了!

They are **too** great!

cf., 他们很厉害!

They are great!

**Example 2**

“真的很 adj.” (really very adj.)

他们真的很棒!

They are **really very** excellent!

cf., 他们很棒!

They are excellent!

**Example 3**

“adj. 啊!” (similar to the usage of “!”)

牛啊!

Superb!!!

cf., 牛!

Superb!

Although the word “辛苦” (painstaking) commonly conveys a negative emotion, its collocation in the corpus all refers to the appreciation for the interpreters’ contribution (see Examples 4 and 5). The commenters believed that deaf interpreters must put more effort than ordinary people because of the hearing loss and the physical exertion of signing. They were moved (“感动”) by the “contagious” (有感染力的) features of the translation, for the expressions of the interpreters were vivid (see Examples 6 and 7).

**Example 4**

哥们**辛苦**了哥们，真的一个人演了一大屋子。

It’s **not easy**, bro. One person played the performance of all people in the show.

**Example 5**

感觉最辛苦的是手语老师，站位也得记。

It feels like the interpretation is the most **painstaking** part, they need to remember the position of each actor as well.

**Example 6**

他们的动作真的很有感染力。

Their body movements are really **contagious**.

**Example 7**

手语老师的表情也好有感染力， 就算不开声音光看画面也好开心好喜庆。

The interpreter’s facial expressions are very **contagious**. I can even feel the happiness from the muted video.

#### Reason exploring

**Figure 9:**
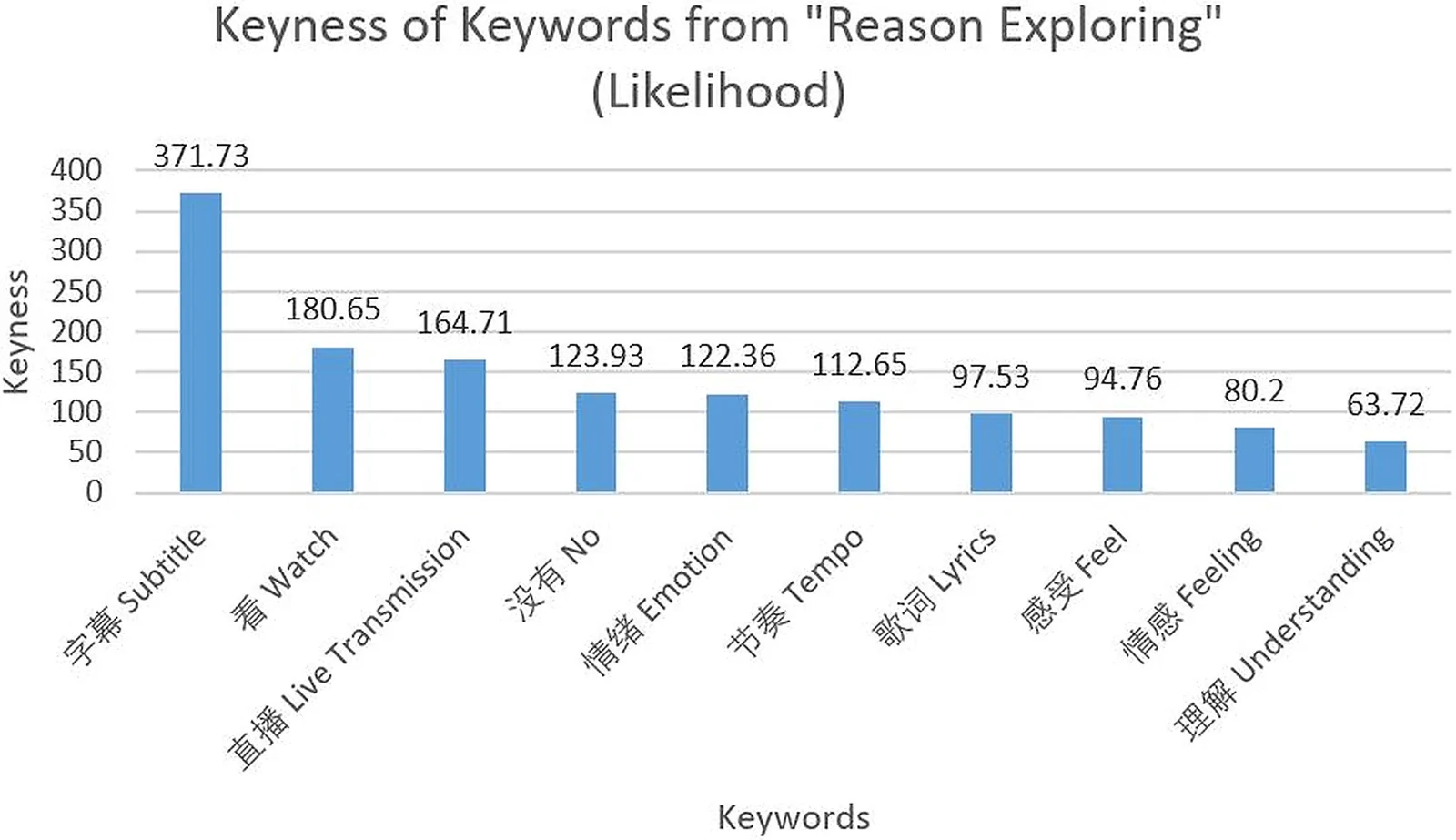
Keyness of keywords in comments of “Reason Exploring” theme.

The “Reason Exploring” theme includes comments questioning the necessity of SL Translation (*n* = 128) and guessing the function of SL Translation (*n* = 142). Keywords of this theme are shown in Figure 9.

There is no shared keyword between the target and referent corpora in this theme. Questions about SLI’s necessity concentrated on the existence of “字幕” (subtitle) and “歌词” (lyrics), assuming that all deaf people can and do understand Chinese characters. Also, believing that oral language could be translated into SL directly in a one word for one sign manner, some users questioned the differences between the interpreters’ mouth shapes and the pronunciation of the lyrics in spoken Chinese (see Examples 8, 9, and 10). However, many Chinese words can only be translated into signs (a combination of gestures, facial expressions, and lip movements) with a similar meaning. These questions revealed the public’s misunderstanding of both SL and deaf people.

**Example 8**

我就好奇一点， 看字幕不应该比看手语快吗， 还不耽误看表演画面。

I am just curious about one thing. Isn’t it true that reading **subtitles** is faster than reading SL? And it doesn’t make people miss the performance.

**Example 9**

我有个疑问，这个手语是给听不见的人展示的吗，可是底下不是有歌词吗?

I have a question. Isn’t the SL provided for deaf people? But I think there are **lyrics** below.

**Example 10**

为什么做的口型和歌词对不上呢?这是手语的一部分吗?

Why does the interpreter’s mouth shape not match the **lyrics**? Is it a part of the SL?

To answer the questions above, some users suggested watching the translation without sound, helping people realize that SL is beneficial for understanding (“理解”) or feeling (“感受”)   the emotion (“情绪”/“情感”) and tempo (“节奏”) of the performances. Additionally, some comments explained that there are performances without (“没有”) subtitles during the live transmission (“直播”), namely the Sketch/Talk Show that involves improvisation. Therefore, the translation allowed deaf people to watch the Gala in real-time, rather than having to wait until a much later date for subtitles to be added before rebroadcasting (Examples 11and 12).

**Example 11**

相声小品都没有字幕的。歌词有字幕但是没有声音的情绪。

There is **no subtitle** for Sketch and Talk Show. Although there is a subtitle for songs, it cannot convey the **emotion** of the songs without sound.

**Example 12**

春晚前有说明， 直播没有字幕， 重播会加上， 所以之前一般都是看重播。加上手语直播可以大家一起看直播了。

There is an explanation before the performance, saying that **no subtitles** will be provided **during the live transmission**, and they will be added before rebroadcasting. Therefore, the SL Translation allows deaf people to enjoy the live performance with us.

#### Association

**Figure 10:**
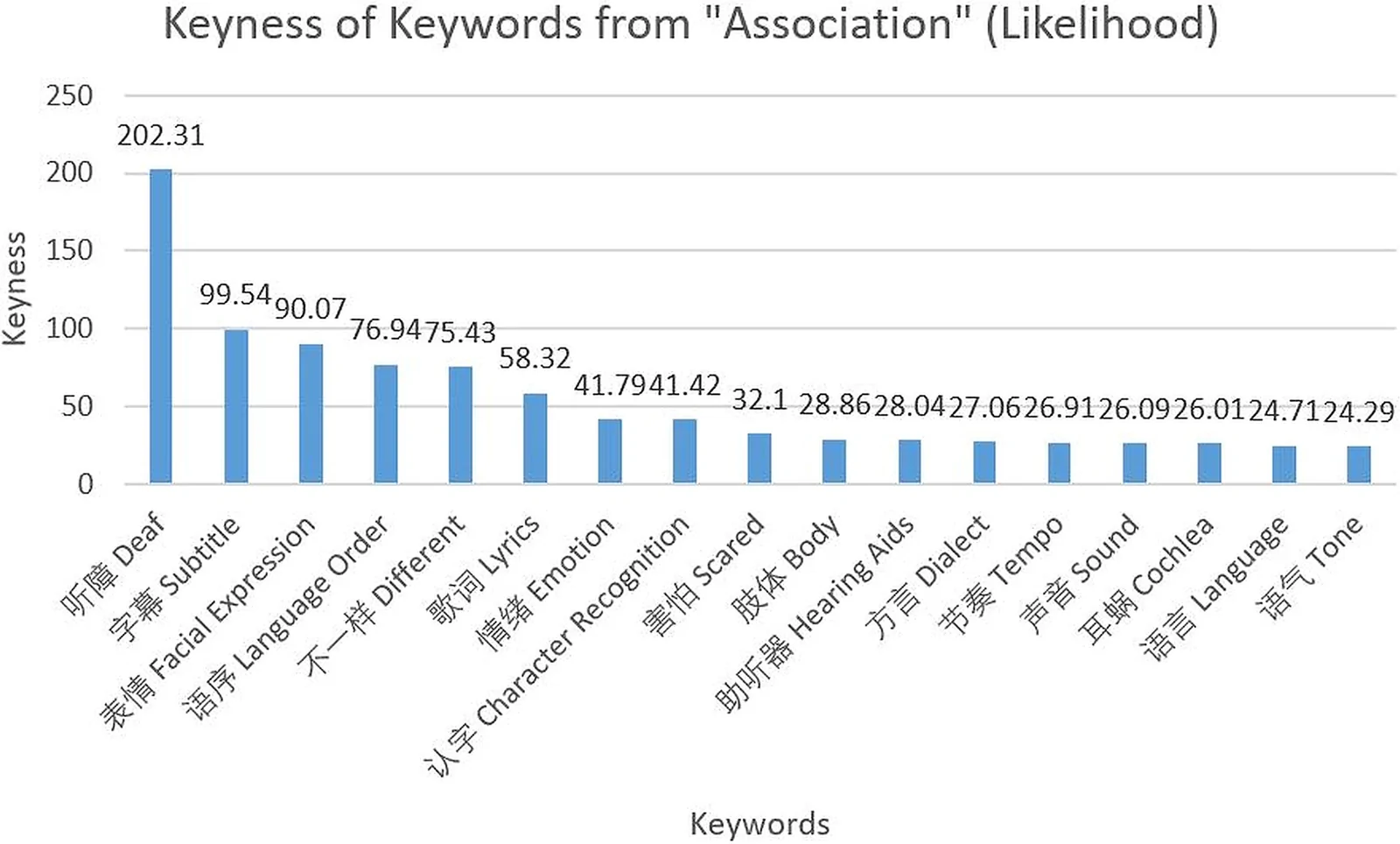
Keyness of keywords in comments of “Association” theme.

The “Association” theme involves comments sharing knowledge about SL or deaf people (*n* = 156), personal experiences of deaf Chinese or people around them (*n* = 37), and other news about SL Translation or deaf people (*n* = 7). Keywords of this theme are shown in Figure 10.

Attitudes toward deaf people varied among the comments (see Examples 13, 14, and 15). Some commenters found deaf people talented at learning, while others took them as ordinary people. Others, however, expressed a negative attitude towards deaf people, scared by their exaggerated sounds and body movements, which is consistent with the traditional public perception of deaf people in Chinese history (see Section 2).

**Example 13**

我以前去给聋哑学校听障的孩子免费教过舞蹈， 他们学得特别快， 一两遍就记住了， 拍子卡的也很准， 非常聪明。

I used to teach dance to **deaf** children in a school for the deaf for free. They learned very quickly and memorized the content by practicing just once or twice. They also kept the beat very accurately, showing their great intelligence.

**Example 14**

我们公司是做助听器的， 日常工作会接触到听障， 其实大家和普通人一样， 就是生活会多一个麻烦。

Our company manufactures **hearing aids**, so we frequently come into contact with individuals who are **deaf** in the course of our daily work. Everyone here is just like an ordinary person, except that they have something inconvenient in daily life.

**Example 15**

我挺害怕听障人的， 因为他们不会说话， 动作就很大， 而且因为听不到， 所以从喉管发出的声音很大他们都不知道。然后他们呈现在我的画面就是:动作和面部很夸张用力， 声音很大， 我没有歧视， 是真的害怕， 我们村就有这种人， 小时候对他又怕又好奇。

I am quite afraid of **deaf people** because they cannot speak, so their movements are exaggerated. And since they cannot hear, they are unaware of the loud sounds coming from their throats. Then the image that comes to my mind is: their movements and facial expressions are exaggerated, and their voices are loud. I don’t mean to discriminate against them, but I am just afraid of them. When I was a child, there were these kinds of people in our village, and I was afraid of and curious about them.

Some deaf people or their family members shared their own experiences. Although some described living a normal life, others experienced discrimination at work, and even at home (see Examples 16, 17, and 18).

**Example 16**

我是中重度听障者， 却偏偏做服务行业， 遇到太多嘲讽和歧视， 心里也很难受， 我强大的心理总能把我自愈。

I am a person with moderate to severe **hearing loss**, but I happen to work in the service industry. I encountered a lot of ridicule and discrimination, which made me feel very uncomfortable. However, my strong mentality always helped me heal myself.

**Example 17**

我一只耳朵听不见， 耳鸣一直伴随二十年了， 以前工作老板说我比较呆， 嫌弃我， 他不知道我听力弱， 我也不想解释。

I am **deaf** in one ear and have had tinnitus for twenty years. My former employer said I was dull and looked down on me. He did not know I had poor hearing, and I did not want to explain to him.

**Example 18**

我父母是聋哑人。从小我就特别不喜欢在外面跟他们交流,因为周围总会有很多让我不舒服的注视的目光。但越长大， 发现其实没有必要在意别人的目光， 自己快乐最重要， 加油!

My parents are both **deaf**. Since I was a child, I did not like to communicate with them outside because there were always many stares around me, making me uncomfortable. But as I got older, I found that there was no need to care about other people’s attitudes. Being happy is the most important. Cheer up!

Additionally, some users shared their knowledge about the differences between SL and subtitles (e.g., “表情” (facial expression) and “语序” (word order)) and the function of SL Translation (e.g., replacing “节奏” (tempo) and “语气” (tone) of the performances with visual-manual modalities to help deaf people understand the emotions), similar to the comments on potential reasons for SL Translation above.

### Study 2

**Figure 11:**
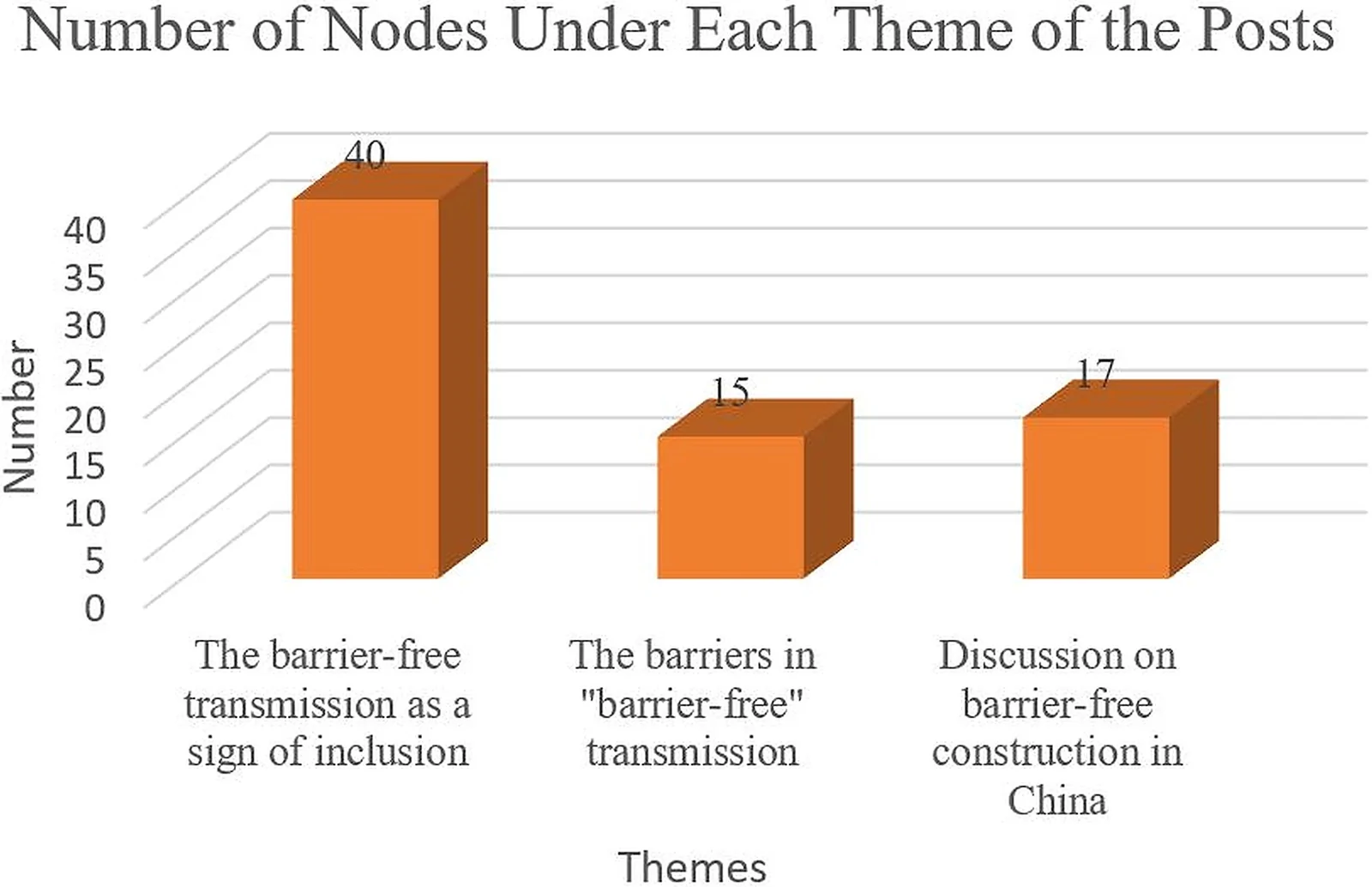
Number of nodes under each theme of the posts (aligning with discussion order in the following parts).

Based on the nine posts from Chinese deaf selected, the following discussions are centered on the three recurring themes extracted from them: (1) barrier-free transmission as a sign of inclusion; (2) the barriers in “barrier-free” transmission; and (3) discussion on barrier-free construction in China. The number of nodes (i.e., coded contents from the posts) under each theme is shown in Figure 11.

Figure 11 shows that positive evaluation of the translation (i.e., as a sign of inclusion) dominated the data. Deaf Chinese generally hold a positive attitude towards the Gala’s SL Translation, though some problems with barrier-free transmission do exist.

#### The barrier-free transmission as a sign of inclusion

Previously, deaf Chinese have found it difficult to engage in the Gala, affecting their socialization with their families and friends (see Excerpt 1). Chinese New Year is a traditional festival for people to get together in China. The Gala plays a significant role in these gatherings, when people watch the performances and communicate with each other during the family reunion. Therefore, the feelings of isolation are exaggerated when deaf people are excluded from participating in these significant moments.

**Excerpt 1**

当聋人看之前的春晚时:打开电视看春晚， 主持人讲话无字幕， 相声小品也没字幕， 我听不见， 不知道别人在说什么。看到别人笑得前仰后合， 我却一脸懵。这时， 我会尴尬地假装跟着笑， 就怕显得自己不合群。感觉自己融入不进去， 有点郁闷也有点难受。(太棒了伞)

Previously, when deaf people watched the Gala, there were no subtitles for the host’s speech, nor the crosstalk and sketch comedies. I couldn’t hear and didn’t know what others were saying. I saw others laughing, but I was confused. Every time I encountered the situation, I would awkwardly pretend to laugh along, for fear of appearing to be out of place. I felt like I couldn’t fit in, and I felt a little depressed and uncomfortable.

The Gala’s barrier-free transmission allowed deaf Chinese to finally understand the performances and participate in family discussions, providing a greater feeling of inclusion (see Excerpt 2). However, it also shows that deaf people often viewed themselves as “局外人” (outsiders) of society. The word “终于” (finally) in Excerpt 3 also revealed that deaf people think this inclusion came “too late” (长颈鹿张涛). One reviewer claimed that the rebroadcasting “has already surprised and satisfied” her (已经很惊喜了, from “有风吹过你的气息”), and she has no more anticipation. The other reviewer also claimed that she was surprised (居然, from “太棒了伞”) by accessible retransmission for the Gala. These words show that deaf Chinese are used to social neglect, indicating the long-standing marginalization of CSL and deaf Chinese in society.

**Excerpt 2**

这次我不再是局外人， 而是真正成为了春晚的一部分， 我能清楚地听到每个小品的笑点， 每首歌曲的深情， 每段相声的幽默。(栾晓煜)

I am not the **outsider** this time, and I have become a part of the Gala. I can clearly “hear” every funny part of the comedies, every emotional part of the songs, and every humorous part of the cross-talks.

**Excerpt 3**

这是我第一次感受到春晚的欢乐氛围， 我很激动， 今年春晚终于再也不寂寞了!(智镜创研)

It is the first time for me to experience the happiness of the Gala, which excited me. I will no longer be lonely this year **(finally)**!

The deaf SLI is another reason for the commenters’ feeling of inclusion. They liked the interpreters’ flexible and exaggerated body movements, as well as their vivid facial expressions (see Excerpt 4).

**Excerpt 4**

陈静和郭家聚两位手语演员， 动作大方、手语大气、表情丰富， 这三点加上音乐唱歌， 合起来非常有节奏感。手语到位!(大巧子)

Chen and Guo, the two SLIs, interpreted with generous gestures, natural sign language, and rich facial expressions. The combination of these three advantages created the tempo. The SL was on point!

To complete the Gala’s SL Translation task, the deaf SLI faced a lot of challenges during both the preparation and the performance, especially in the interpretation of singing. To match the translation with the tempo of the original songs and artistic aesthetics, Guo Jiaju, the deaf SL songwriter, explained that he revised the translations many times before performance. Other deaf interpreters relied on portable beat vibrators and commanders to memorize the tempos in preparation (Disability in Chinese, 2025). Their persistence and final success inspired the deaf online commenters, who also considered the deaf SLI’s participation in the Gala as a symbol of deaf people’s role-change in society, from “旁观者” (bystanders) to “参与者” (participants) (大巧子). This verifies and deepens Wang & Li’s “自己人效应” (The Phenomenon of Preferring People on One’s Own Side).

Generally, the deaf online commenters took this accessible retransmission as a starting point for social progress on the recognition of CSL and deaf Chinese. They believed that there would be more barrier-free constructions for them in the future, and Chinese society would become increasingly inclusive overall.

#### The barriers in “barrier-free” transmission

Although all commenters described looking forward to the accessible retransmission before the Gala, some of them expressed disappointment with the access once it had been broadcast, indicating the potential of “illusion of inclusion” abovementioned.

To view the Gala rebroadcast, complicated technological procedures were required for both deaf and blind users (see Excerpts 5 and 6), but there was no specific instruction on the procedures. It was necessary for individuals to first download and install an extra application (“China Media Group Mobile” for SL, and “Cloud Player” for broadcasting), which is impossible for people without digital devices and those who were not taught how to do this prior.

**Excerpt 5**

在哪看呢?下载APP——央视频或者央视新闻。下载之后， 在手机上看也可以， 在平板上看也可以， 电视下载也可以看， 或者手机投屏电视也可以， 方法很多。我相信我们聋人朋友都知道怎么操作。如果老年聋人不知道怎么操作， 可以问问身边的孩子， 让他们帮忙准备好。(大巧子)

Where can we find the transmission? Download the application “CCTV Video” or “CCTV News.” After downloading, you can watch it on your phone, tablet, TV, or by casting the phone screen to the TV. There are many ways. I believe our deaf friends know how to operate it. If the elderly deaf people don’t know how to operate it, they can ask their children around them to help prepare it.

**Excerpt 6**

在这个了解的过程中， 我真的觉得观看无障碍解说， 让我觉得障碍重重。首先， 我是通过朋友了解到这件事情的， 我本身根本不知道怎么去观看， 然后我好不容易搞清楚了， 是需要下载一个云听的APP， 于是我就去下载了。下载好之后呢 我又发现这个云听打开分类之后， 我是没有办法正常用我的软件获取到分类的， 我只能通过屏幕识别这些辅助手段。就相当于我们特别激动地区找到一个宝箱， 或者找到了一把锁， 想要打开它， 但是我发现没有钥匙， 就差临门一脚了， 然后我们只能去找开锁公司， 那这个过程真的就是垃圾体验了。(晴晴本情)

However, during the process of understanding, I felt that watching barrier-free commentary was full of barriers. First of all, I learned about this barrier-free transmission from a friend. I did not know how to watch it myself. Then I finally figured out that I needed to download a cloud listening app, so I downloaded it. Afterwards, I found that I could not get the category normally with my software. I could only use the screen to identify these auxiliary means. The experience is like when we were very excited to find a treasure chest or a lock and wanted to open it, but found that we did not have the key, and it was just one step away. Then what we could do was to go to the locksmith company, and this process was a rubbish experience.

When watching the retransmission, the “unclear screen” and “delay in transmission”(see Excerpts 7 and 8) affected the commenters’ experiences. Some deaf people also claimed that they could not fully understand the SL in the transmission, and the contents in the broadcasting for blind people were not complete enough for them to imagine the scene of some performances (see Excerpt 9). The so-called “real-time, accessible retransmission” was not borne out in reality.

Clark (2014) claimed that the accessible services at present were provided under the assumption that “there is only one world”, i.e., the world of hearing and sighted people. They are trying to include the disabled in their perspective (at the providing end), instead of considering the real needs of the disabled in the receiving end. Those complicated procedures and incomprehensible SL strengthened deaf people’s sense of powerlessness. Is this true inclusion? Or just an “Illusion of Inclusion”?

**Excerpt 7**

开始观看春晚时， 手机下方有一个大方框屏幕正在进行手语翻译， 屏幕中间有滚动字幕， 但上面小方块屏幕太小了， 看不清春晚画面， 在大电视上看春晚看看明星有哪些， 在春晚、手语、字幕之间来回看， 时间一长， 我的眼睛好忙， 忙得看不过来了。(大巧子)

When I started watching the Gala, there was a large square screen at the bottom of my phone that was doing SL Translation, with scrolling subtitles in the middle of the screen, but the small square screen above was too small to see the content of the Gala clearly. I had to watch the Gala on a big TV screen to see which stars were on, and went back to my phone for the SL Translation, switching among the Gala, SL, and subtitles continuously. After a while, my eyes were so busy that I couldn’t keep up.

**Excerpt 8**

因为这个是障碍转播节目， 我和我的健听朋友们其实是不在一个频道的， 会有延时性， 不能即使共同讨论春晚的感受， 所以我还是会有些落寞感。(有风吹过你的气息)

Since this is a barrier-free broadcast, my hearing friends and family members were not on the same channel as I. There was a delay, and we couldn’t discuss our feelings about the Gala together in time, which still made me feel a little lonely.

**Excerpt 9**

它是很关键的部分才会给一些解说， 但是这样就会导致一个问题， 就是有些地方依然获取不到内容。比如说我刚开始看那个小品的时候， 看那个《白蛇传》的小品， 有很多动作我不知道、搞不清楚是在做什么。我也比较理解， 他们想要让我们更沉浸式地去观看这个影片， 所以就没有在空隙中插播解说， 但是我觉得我们是不是可以更好地去审阅这个无障碍解说地稿件， 然后尽可能地利用这些空档来为我们解说。我觉得这是一个可以改进的方面。(晴晴本情)

I found out that it was a very critical part that only some of the commentary was given, but this would lead to a problem, that is, at some point I still could not get the content. For example, when I first watched a comedy, the comedy about the Legend of the White Snake, there were many actions that I didn’t know and couldn’t figure out what they were doing. I also understand that they want us to watch the film more immersively, so there is no commentary in the gaps for interruption, but I think we can better review the manuscript of the barrier-free commentary and use these gaps as much as possible to explain to us. I think this is an aspect that can be improved.

Nevertheless, all the commenters showed their tolerance of the issues mentioned above (see Excerpts 10 and 11), since it was the first time for the Gala to try out accessible retransmission. They held a positive attitude towards the future of Chinese barrier-free construction.

**Excerpt 10**

虽然说还是有一些不足的地方， 但是这个事情是特别重要的一步。我们聋人群体的信息无障碍， 迈出了很大的一步。有一些不足是正常的， 因为是第一次嘛。我相信以后肯定会有越好越好的空间和未来。重要的是这个事情做出来了， 从无到有， 而且还是那么大的一个平台， 这个意义太大了， 给以后的好多不同的领域都提供了一个示范作用。(DUDU杜银铃)

Although there were still some shortcomings of the SL Translation, this has already been a significant step toward barrier-free construction for deaf people. It is normal to have problems at first. But I believe the future will be better and better. What’s important is that it has been accomplished, from nothing to something, on such a huge platform. This is of great significance, providing a model for other fields in the future.

**Excerpt 11**

万事开头难嘛!希望后面春晚可以做得更好!(有风吹过你的气息)

The first step is always the hardest. I hope the following Gala can behave better than this year!

#### Discussion on barrier-free construction in China

Apart from the feedback on the Gala’s “inaccessible” retransmission, the users also provided suggestions to Chinese barrier-free construction on three levels: (1) forms of translation; (2) understanding of barrier-free construction; and (3) deaf people’s social issues.

Firstly, deaf viewers of the Gala could not fully understand the Gala’s SL Translation, and they needed subtitles as supplements, emphasizing the necessity to consider the various linguistic preferences of deaf people (see Section 2). Both SL Translation and subtitles are necessary for deaf people to understand TV programs.

Regarding the AI interpreter, although it was used during the Beijing Winter Olympic Games (Xiangli, 2023), it lacks facial expressions and natural gestures, which are crucial for understanding SL. This not only affects the public perception of real CSL but also magnifies deaf people’s “distantism.” Consequently, it is rejected by deaf Chinese for providing SL translation during the Gala (see Excerpt 12). To achieve true inclusion for deaf Chinese, the suggestions mentioned above should be taken seriously.

**Excerpt 12**

我看小品的时候， 需要有字幕和内容搭配， 我才知道小品讲什么内容。去掉字幕只有手语， 我只能看得一知半解。这是春晚无障碍转播唯一的缺点。

…

中国残疾人杂志社公众号发表了一篇文章， 文章说， 以前最早的方案是有人想用手语+AI， 合成一个“手语数字人”做手语翻译， 导演组去问听障群体的意见， 发现很多听障朋友反对， 不喜欢用手语数字人。为什么?手语数字人站着打手语， 手势标准正确， 但是缺了什么呢?没有表情。你光看那个手语， 不看表情， 我们聋人看不懂。我们聋人要看什么?手语+动作+表情， 才能看懂手语。(大巧子)

Without subtitles, there was only sign language, and I could only understand half of it. This is the only shortcoming of the accessible transmission of the Spring Festival Gala.

……

The official account of China Disabled Persons Magazine published an article, saying that the earliest plan was to use sign language + AI to synthesize a “sign language digital human” as a sign language interpreter. The director team asked the deaf group for their opinions and found that many deaf friends opposed and did not like the sign language digital human. Why? The sign language digital human stood and signed, and the gestures were standard and correct, but what was missing? No facial expression. If you only look at the sign language and not the facial expression, we deaf people can’t understand it. What do we, deaf people, want to see? Sign language + action + facial expression, to understand the sign language.

Secondly, some commenters believe that barrier-free construction is beneficial to everyone in need (e.g., when people are in a noisy environment or with poor sight). Moreover, commenter晴晴本情  criticized both the forms and the procedures of gaining access (see Excerpt 13). The retransmission needed to be accessed on another platform, instead of being together with the TV program, creating the feeling of isolation among the disabled. She hoped she could enjoy the TV program together with her friends and family members naturally, with no barrier to the “barrier-free” construction, which is essential to true inclusion.

**Excerpt 13**

大家可能会觉得， 跟我关系不大， 我又没有障碍。那其实呢， 我们也是能用到的， 因为比如说在我们比较嘈杂的环境中， 我们也是需要字幕的。或者说呢， 我们年老了， 或是视力有下降的时候， 我们也会需要这个东西去帮我们获取内容。……那这次转播的感觉呢， 其实有些地方会让我觉得有被隔离， 就是我需要去通过听广播， 我需要通过下载新的APP， 而且它无障碍还不好， 而不是一个传统的方式， 比如说我就是很自然地在看春晚的过程中就有无障碍， 我是需要去被隔离到一个另外的地方去观看的。就像小的时候， 可能一些落后的地方会觉得女人是不能上桌的， 就是这种感觉。所以我希望我以后看到的无障碍的解说， 是不需要很有障碍地去获取它， 我可以很自然地去观看它， 在享受万家灯火的同时， 也享受到了无障碍， 我觉得这才是我想要的样子。(晴晴本情)

Everyone has a chance to need barrier-free transmission, because, for example, in a noisy environment, we also need subtitles. Or, when we are old or our eyesight is declining, we will also need this thing to help us get content. …… As for the feeling of this broadcast, some places make me feel isolated, that is, I need to listen to the radio, I need to download a new APP, and it is not barrier-free, rather than a traditional way that I can naturally have barrier-free when watching the Spring Festival Gala. I need to be isolated in another place to watch it. The feeling is just like when I was a kid, some backward places might think that women can’t sit at the table. There, I hope that the barrier-free explanations I see in the future will not require a lot of obstacles to access, and I can watch them easily, enjoying the lights of thousands of homes while also enjoying barrier-free access. I think this is what I want.

Thirdly, based on Terje Basilie’s words (see Section 2.4), behind the perception of a language, it is the social situation of its users. Besides talking about the accessibility of the SL Translation, some users admitted that the current situation of deaf Chinese is still non-positive. They still face many challenges in daily life, experiencing inconvenience at work, in schools, and in other public places. To help deaf people live a better life, in addition to the barrier-free transmission of TV programs, the improvement of barrier-free construction based on CSL acceptance and standardization, as well as the development of social equality, are necessary (see Excerpt 14).

**Excerpt 14**

当然， 真正实现包容友好的残疾人社会， 还有很长的路要走， 在现实生活中, 我们仍然面临就业歧视、沟通难题、无障碍设施不符等诸多挑战。(栾晓煜)

Of course, there is still a long way to go before the establishment of an inclusive and disability-friendly society. In real life, we, the deaf people, still face challenges like discrimination, difficulties in communication, and incomplete accessible infrastructure.

Also, the popularization of CSL is not easy. The commenters believed the influence of celebrities could be a good way to spread CSL (see Excerpt 15). One commenter mentioned Lay Zhang, a singing star with over 50 million followers on Chinese social media^8^ (June 2025). He played the role of a deaf father in a Chinese movie, Mumu, and learned CSL from deaf Chinese for this role. Afterwards, he started to care about the lives of Chinese deaf people and became the first Chinese singer to provide SL Translation and special services for deaf people in his concerts. He even communicated with deaf people in SL during public media events, allowing his followers to know more about deaf people and SL in China.

**Excerpt 15**

春晚无障碍转播的时候， 张艺兴上台和主播聊天， 他还用了手语。我觉得他作为一个流量明星, 可以帮我们宣传手语。比如聋人文化上各种误区,靠我们这些聋人宣传的力量, 还是不如流量明星传播来的快。所以张艺兴帮我们宣传了好多好多， 特别好。本来手语科普是任重道远的事情， 有他这样的明星宣传， 会快一些。(DUDU杜银铃)

During the barrier-free transmission of the Gala, Lay Zhang (a Chinese singing star) talked with the hosts on the stage in SL. I think he can help us promote SL as a social media star. For example, when talking about the misunderstanding of Deaf Culture, the power of propagation by us deaf people is not as strong as that spread by online celebrities. Therefore, it is good that Lay Zhang has helped us spread the SL a lot. It should be admitted that popularizing sign language is a long and arduous task. With a star like him promoting it, it will be much faster.

## Discussion

Based on Loh’s (2025a) theory that language ideology can be extracted from both explicit and implicit public discourses, this research combined the corpus-assisted and qualitative analyses of online discourses about the Gala’s SL Translation to extract the public ideology of CSL (Studies 1 and 2). It was found that both the general public and deaf viewers mainly showed a positive attitude towards the Gala’s SL Translation, though the underlying reasons for these views and attitudes varied.

In Study 1, the general public was mainly attracted by the novelty (Ku & Chen, 2007) of SL Translation (cf. the general non-positive statements in the referent corpus), with many people expressing that they were exposed to SL for the first time, especially when combined with aesthetic performances. This, to some extent, revealed the shortage of SL Translation availability in China despite the national policies encouraging CSL usage and a high number of TV channels now providing SL Translation (see Sections 2.2 and 2.3). Also, deaf SLI with natural SL and vivid facial expression made a difference in the public acceptance of CSL as well, compared with the traditional hearing SLI in other Chinese TV programs (see Section 2.3).

In Study 2, the deaf commenters’ positive feedback came from their belief that the Gala’s SL Translation is the starting point of the recognition of Deaf Culture and CSL at the national level. This, in turn, indicates the long-term neglect of deaf people’s needs previously. The commenters preferred the expressions of the deaf SLI to the hearing SLI or AI SLI, regarding deaf SLI as a symbol of inclusion and clarity. This aligns with Wang and Li’s (2015) observation of “自己人效应” (The Phenomenon of Preferring People on One’s Own Side).

However, negative information exists in comments from both the general public and the deaf commenters.

Due to their hearing-centered perspectives, as well as limited knowledge about Deaf Culture, the public comments indicate severe misunderstandings of CSL and deaf Chinese. They assumed that deaf people all read and understand Chinese characters, and therefore subtitles. Yet in China, the dominant O-A teaching approach and limitation in higher education resulted in the poor education of deaf children (see Section 2).

Moreover, text reading requires the language competencies of spoken language, which have various grammatical features from SL. However, subtitles are still useful for disambiguating meanings when the SL Translation is not clear (see Excerpt 12). Therefore, both SL and subtitles are significant for deaf people, if they are effective in conveying information to diverse deaf audiences (e.g., deaf people of various ages, regions, and educational levels, Hodge & Goswell, 2023).

Deaf people also shared their personal social experiences in the online posts. These include suffering from discrimination at work and even at home, showing that the “secondary disability” (Dongdong, 2020) of deaf people is still severe nowadays. Deaf people shared that they are not considered or understood by the public, which includes not only ordinary people but also their family members. This can be a result of the general neglect of people’s needs (Zimu, 2020) and traditional negative ideas about deaf people in China (Zheng, 2022).

The SL Translation in the Gala didn’t address common problems seen in other TV programs (e.g., Cao, 2018; Shen, 2013), like transmission delays, unclear screens, unreadable SL, and lack of subtitles. These issues disappointed deaf viewers, increasing their sense of isolation and reflecting the link between language status and user identity (Loh, 2025b). To achieve the goal of Chinese barrier-free transmission, i.e., ensuring deaf people’s equality and access to information (Li, 2013), improvements are needed.

Generally, it can be indicated that the present situation of Chinese Deaf Culture is affected by policies, programs, and goals established by hearing people to “fix the broken people” (Lytle et al., 2005).

Therefore, the deaf commenters suggested a national reconsideration of accessibility. Apart from the barrier-free transmission, other barrier-free access in China remains inaccessible to some extent for deaf people, as it is often established from a “hearing- or sight-centered” perspective (Clark, 2014). This results in the “illusion of inclusion” and “distantism.” To address this issue, improvement is necessary at a system-level, about the political and cultural factors that affect and perpetuate the barrier-free access (Haualand et al., 2022), besides focusing on the specific SLIS at a micro level. Also, increasing the quantity of access is insufficient; the quality of access must be considered seriously.

**Figure 12:**
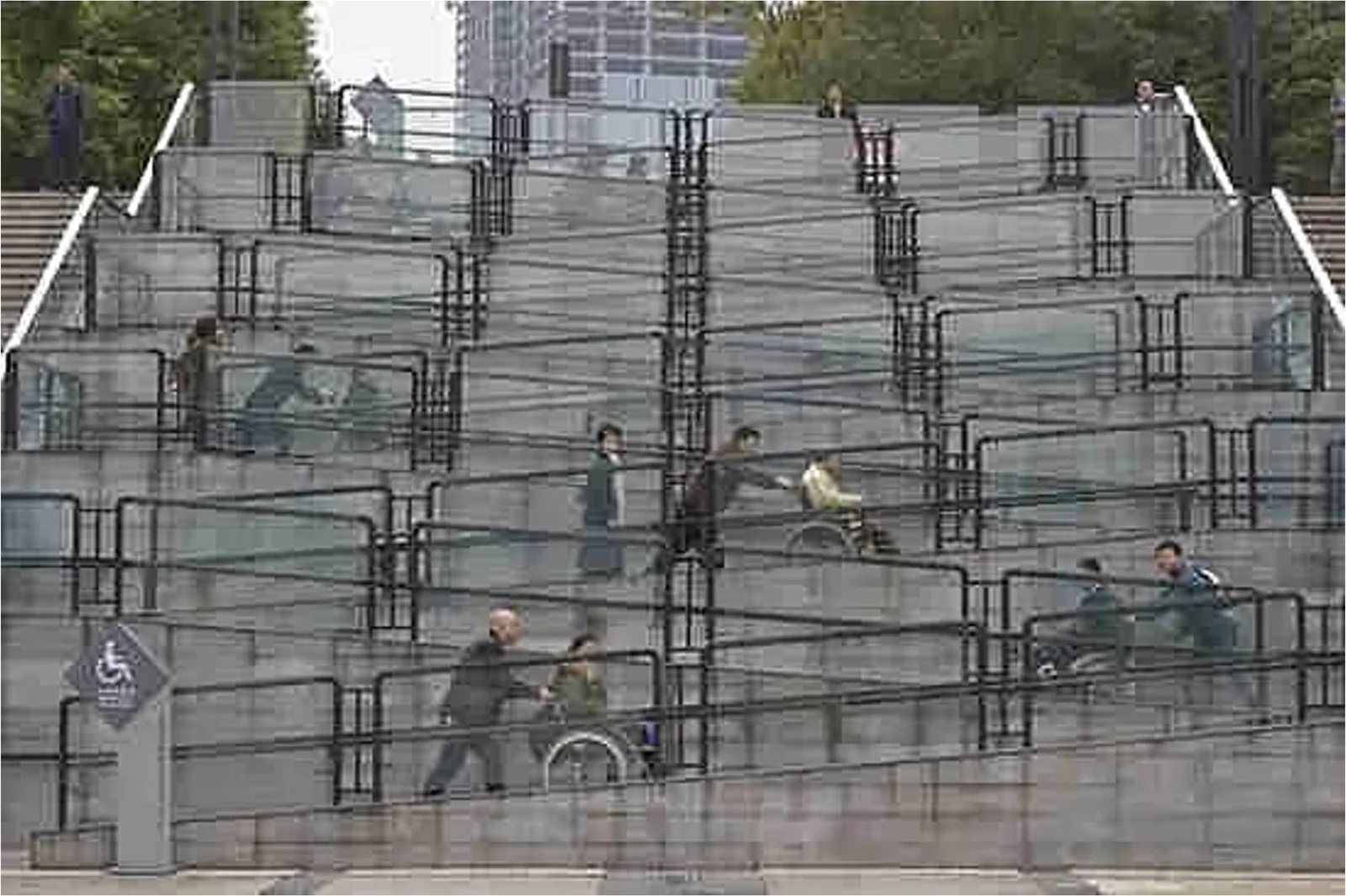
Challenge of going uphill (cited from Zimu, 2020).

The picture below (Figure 12), titled “Challenge of Going Uphill”, shows the difficulty of going uphill in wheelchairs. Accessible slopes allow access but require more energy and time for wheelchair users and their companions. Zimu (2020) compared this to deaf Chinese confronting barriers to accessing “barrier-free” services, like learning Chinese characters and SC, before understanding TV translations or buying SLIS for communication.

Is this the goal of Chinese barrier-free construction? Who should be the one to decide what is “accessible,” and how can deaf people feel “more accessible”? To answer these questions and achieve real accessibility among deaf Chinese, their voices should be included (Haualand et al., 2022).

## Conclusion

To understand hearing and deaf Chinese people’s perception of the SL Translation in the 2025 CMG Chinese New Year’s Gala, this research employed a combination of quantitative and qualitative methods for analyzing comments online. Using data from a novel corpus of online comments from RedNote and feedback from deaf/blind Chinese, the analyses revealed a generally positive perception of the SL Translation and the SLI, viewing it as a sign of national development in barrier-free construction. However, these analyses also revealed that a lot of work is still required before establishing a truly inclusive and equal society for the deaf. The shift in hearing-centered perspectives, as well as the legal recognition and standardization of CSL, are crucial for the future development of Chinese barrier-free constructions.

## Footnotes

1 《国家手语和盲文规范化行动计划(2015–2020)》  https://www.pkulaw.com/chl/cb14b6e8d6e49f59bdfb.html?way=listView

2 《国家手语和盲文规范化行动计划(2021-2015)》   https://www.pkulaw.com/chl/bda5de8a12ccf280bdfb.html?way=listView

3 《关于加快在特殊教育学校推广国家通用手语和国家通用盲文的通知》https://www.cdpf.org.cn/zwgk/zcwj/wjfb/8c873977bafd46e0b87c42669c8325a3.htm

4 《关于进一步加强残疾人广电视听公共文化服务工作的通知》 https://www.nrta.gov.cn/art/2025/1/27/art_114_70136.html

5
https://wfdeaf.org/the-legal-recognition-of-national-sign-languages/

6
https://bcc.blcu.edu.cn/

7
https://www.questmobile.com.cn/research/report/2000767092954075138

8
https://weibo.com/n/%E5%BC%A0%E8%89%BA%E5%85%B4.

## Appendix A: Scraped Videos

These data span January to May 2025 and were collected in May 2025.

**Table:**

	Creator	Time	Title	Likes	Comments
1.	火车侠	2025/02/01	[春晚手语] 《妥妥的》手语简直就是街舞! https://www.xiaohongshu.com/explore/679db158000000001801a8c6?xsec_token=ABfTy5WWYOH6xNURuGar4PIi9X1mUyDk1P7fQ9jX3nkkk=&xsec_source=pc_collect	140,000	394
2.		2025/01/28	孤勇者春晚版——无障碍转播 https://www.xiaohongshu.com/explore/6799373100000000180100f3?xsec_token=AB2MaKQxbEkVPbmojeilIYavd6GCdkf_N_2RCTKtxDwzw=&xsec_source=pc_collect	190,000	622
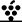
3.	大巧子	2025/01/30	春晚无障碍转播观后感 https://www.xiaohongshu.com/explore/679b4f7e0000000018013184?xsec_token=ABXdp41rwQFow_oaazTyoc1Vd9lzfla1bz2rywN6h000U=&xsec_source=pc_collect	278	43
4.	小央视频	2025/01/14	总台春晚首次推出无障碍转播 https://www.xiaohongshu.com/explore/678639aa0000000020022199?xsec_token=ABpsA2NWBMq9MfNSsku6gEyZuP-ooMO4d_yfouCtHN2UU=&xsec_source=pc_collect	6,926	277
5.	橙子Bro	2025/02/06	《玉盘》听障版无障碍转播 2025 年春晚 https://www.xiaohongshu.com/explore/67a4cbc6000000001800c8c3?xsec_token=AB0Mq3fwYG7rWIVSqUb3bRODpYmQ61exeYubUnXCL6PF0=&xsec_source=pc_collect	9,844	473
6.	小酒窝	2025/02/02	《登高》春晚手语版无障碍版 https://www.xiaohongshu.com/explore/679faa490000000029037084?xsec_token=ABzJut9pIf1v_tbPzLsegcetDfEDfFfN4DIuddRfEQ8R4=&xsec_source=pc_collect	1,720	102
7.	庄胜春	2025/01/28	他们是听障女孩和男孩， “唱“出最炸的孤勇者 https://www.xiaohongshu.com/explore/67984c870000000017039de6?xsec_token=ABDFnW_-izb5a-iogROSzruvJXN_FxMFEkPm5uIlx-MPE=&xsec_source=pc_collect	1,770	113
8.	有风吹过你的气息	2025/01/28	听障者对春晚无障碍转播的感受 https://www.xiaohongshu.com/explore/6798f11d0000000018011b92?xsec_token=ABDFnW_-izb5a-iogROSzrupkr2MspC4WWvm33kHDhpzE=&xsec_source=pc_collect	21,000	264
9.	我只磕一对吗?我磕到多对的	2025/02/01	原来手语版是这样的!!! https://www.xiaohongshu.com/explore/679e0a88000000002901ee9a?xsec_token=ABwKm0_0LAVpyw_x4PXBhgxnnp3d6KMYD1Jzbxp3T5SQA=&xsec_source=pc_collect	3,318	220
10.	红星新闻	2025/01/18	春晚首次无障碍转播听障版彩排， 台下的导演看哭了 https://www.xiaohongshu.com/explore/678b30c6000000001802ed42?xsec_token=ABYJ6tXGstt6OfyHReoY8HRVaz7y6GyoGoYBm1f68pL7U=&xsec_source=pc_collect	690,000	1,832
11.	简岩的夏天	2025/01/29	第一次发现春晚还有无障碍(手语)版本直播 https://www.xiaohongshu.com/explore/6798fdc50000000018004c29?xsec_token=ABDFnW_-izb5a-iogROSzrugqDYjZdLhn_hLGkb64uqso=&xsec_source=pc_collect	3,532	105
12.	春晚	2025/02/04	手语版《一起China Fun》， 快来一起玩儿! https://www.xiaohongshu.com/explore/67a1cae60000000018010cd7?xsec_token=ABljZ3zeyBScd5IDULEfD-ZmjsgMTowDyG3Fnhd_LSKlg=&xsec_source=pc_collect	2,414	302
13.	春晚	2025/02/02	全网都在找的手语版《世界赠予我的》来啦! https://www.xiaohongshu.com/explore/679f783a000000002900e33a?xsec_token=ABzJut9pIf1v_tbPzLsegcelx8hUp5cca4ku7lde0i1NY=&xsec_source=pc_collect	11,000	299
14.	春晚	2025/01/12	让爱无碍!总台春晚首词推出无障碍转播❤️ https://www.xiaohongshu.com/explore/67834a5d0000000017038d82?xsec_token=ABMZ8BNeONQred2_6yHdpIZUxmmOwSolSJOE8RrdXKYh0=&xsec_source=pc_collect	19,000	202
15.	春晚	2025/02/02	手语版《假如》， 带你静静回望来时路~ https://www.xiaohongshu.com/explore/679f799d000000002900e78e?xsec_token=ABzJut9pIf1v_tbPzLsegcel6EeQmvBdBLTfNxVztR-to=&xsec_source=pc_collect	586	33
16.	春晚	2025/03/03	手语版《玉盘》大气演绎振奋人心 https://www.xiaohongshu.com/explore/67c598e4000000000e0045d5?xsec_token=ABrBiR8LEojtaxTEDOctYsgCO4MXxry_KYMGvFOOLHW1w=&xsec_source=pc_collect	3,092	98
17.	春晚	2025/02/02	手语版《我的家》， 感受不一样的精彩! https://www.xiaohongshu.com/explore/679f78b100000000290149f3?xsec_token=ABzJut9pIf1v_tbPzLsegcev74XByIxyhpu_Md4QyHRXs=&xsec_source=pc_collect	829	39
18.	小高(见过凤凰传奇版)	2025/02/02	春晚无障碍转播， 太棒了吧!凤凰传奇真费手语老师 https://www.xiaohongshu.com/explore/679f62b80000000028036f1a?source=webshare&xhsshare=pc_web&xsec_token=ABIJ5n8togkzEasT2lBgmmiPs5aDvcfyP2zYPCB36DXao=&xsec_source=pc_share	1,560	483
19.	春晚	2025/02/01	手语版《栋梁》的全新演绎， 感染力超强! https://www.xiaohongshu.com/explore/679d946700000000180185d5?xsec_token=ABfTy5WWYOH6xNURuGar4PIpw-fOi7GEHlt2KHb-Bd96U=&xsec_source=pc_collect	1,435	91
20.	春晚	2025/02/02	当戏曲遇上手语， 全新的打开方式 get! https://www.xiaohongshu.com/explore/679f7abe000000002902e4dd?xsec_token=ABzJut9pIf1v_tbPzLsegcekPIa6qIwzYyHmkUAQhfJis=&xsec_source=pc_collect	623	31
21.	阿布布酱	2025/01/30	2025春晚无障碍版本 0713《登高》 https://www.xiaohongshu.com/explore/679b4f960000000018009419?xsec_token=ABXdp41rwQFow_oaazTyoc1YyL6urraJyohEWqYN13iE0=&xsec_source=pc_collect	1,196	54
22.	手语翻译荆小笛	2025/01/29	https://www.xiaohongshu.com/explore/6799c1ba00000000290137c0?xsec_token=AB2MaKQxbEkVPbmojeilIYak-gs7x1_AEqPG295KkHPuQ=&xsec_source=pc_collect	1,082	59
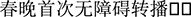
23.	中国聋人	2025/02/03	手语歌天花板级的rap表演—《栋梁》 https://www.xiaohongshu.com/explore/67a03d7000000000290107dd?xsec_token=AB8tvNXH7hTNcs5BiYVfLotCYeVfSstx2vVD5P4f3-WGU=&xsec_source=pc_collect	7,797	291
24.	彬彬	2025/01/31	手语主持 https://www.xiaohongshu.com/explore/679c3d2c0000000018010ef9?xsec_token=ABh9WSGoq_V9Kmq0GqDafZ4VTgqa57OxBOyg6qyNtLrIc=&xsec_source=pc_collect	1,003	78
25.	太棒了伞	2025/02/17	聋人听不见怎么看春晚? https://www.xiaohongshu.com/explore/67a20001000000002900f015?xsec_token=AB3cUUtKa_TyBOx7nLtXoyEUT3Vkr8rxSYy2_Pi9RS7uE=&xsec_source=pc_collect	479	33

## Appendix B: Transcribed Posts

These data span January to May 2025 and were collected in May 2025.

**Table:**

	Creator	Time	Title	Likes	Comments
1.	DUDU 杜银铃	2025/01/31	【DuDu分享】春晚无障碍转播看到了小马 https://www.bilibili.com/video/BV1NGFReWEU7/?spm_id_from=333.337.search-card.all.click&vd_source=fa7faca22e2830ae0b43d11127831665	111	9
2.	大巧子	2025/01/30	春晚无障碍转播观后感 https://www.xiaohongshu.com/explore/679b4f7e0000000018013184?xsec_token=ABXdp41rwQFow_oaazTyoc1Vd9lzfla1bz2rywN6h000U=&xsec_source=pc_collect	278	43
3.	大巧子	2025/01/27	聋人的春晚:春晚无障碍转播 https://www.xiaohongshu.com/discovery/item/67972cf50000000018011c7f?source=webshare&xhsshare=pc_web&xsec_token=AB39ClRyRDjDp0_B1uvtZ5bmK1IGHy_zz3bz5v5-695m0=&xsec_source=pc_share	77	10
4.	栾晓煜	2025/01/28	春晚无障碍转播太棒了!	60	6
5.	太棒了伞	2025/02/17	聋人听不见怎么看春晚? https://www.xiaohongshu.com/explore/67a20001000000002900f015?xsec_token=AB3cUUtKa_TyBOx7nLtXoyEUT3Vkr8rxSYy2_Pi9RS7uE=&xsec_source=pc_collect	479	33
6.	有风吹过你的气息	2025/01/28	听障者对春晚无障碍转播的感受 https://www.xiaohongshu.com/explore/6798f11d0000000018011b92?xsec_token=ABDFnW_-izb5a-iogROSzrupkr2MspC4WWvm33kHDhpzE=&xsec_source=pc_collect	21,000	264
7.	长颈鹿张涛	2025/01/24	春晚大变化!无障碍转播!GREAT NEWS!!! https://www.xiaohongshu.com/discovery/item/6792722f000000002901d59c?source=webshare&xhsshare=pc_web&xsec_token=ABAgg1zsSWJezJpOPBrgnhFKA4k2LM8qh-Njt4wYOIAJ4=&xsec_source=pc_share	260	41
8.	智镜创研	2025/05/04	春晚你也是好起来了! https://www.bilibili.com/video/BV1vBVKzZEpV/?spm_id_from=333.337.search-card.all.click&vd_source=fa7faca22e2830ae0b43d11127831665	3	0
9.	晴晴本情	2025/01/30	盲人眼中的春晚是怎样的? https://www.xiaohongshu.com/discovery/item/679b2703000000002503eab9?source=webshare&xhsshare=pc_web&xsec_token=ABXdp41rwQFow_oaazTyoc1f29b01jFPSpwFIxodBwtSw=&xsec_source=pc_share	1,675	121
